# Techno-Economic Assessment of Electromicrobial Production of *n*-Butanol from Air-Captured CO_2_

**DOI:** 10.1021/acs.est.3c08748

**Published:** 2024-04-15

**Authors:** Jeremy David Adams, Douglas S. Clark

**Affiliations:** † Department of Chemical and Biomolecular Engineering, University of California, Berkeley, Berkeley, California 94720, United States; ‡ Molecular Biophysics and Integrated Bioimaging Division, Lawrence Berkeley National Laboratory, 1 Cyclotron Road, Berkeley, California 94720, United States

**Keywords:** electromicrobial production, electrofuel, e-fuel, carbon capture and utilization, biofuel, TEA

## Abstract

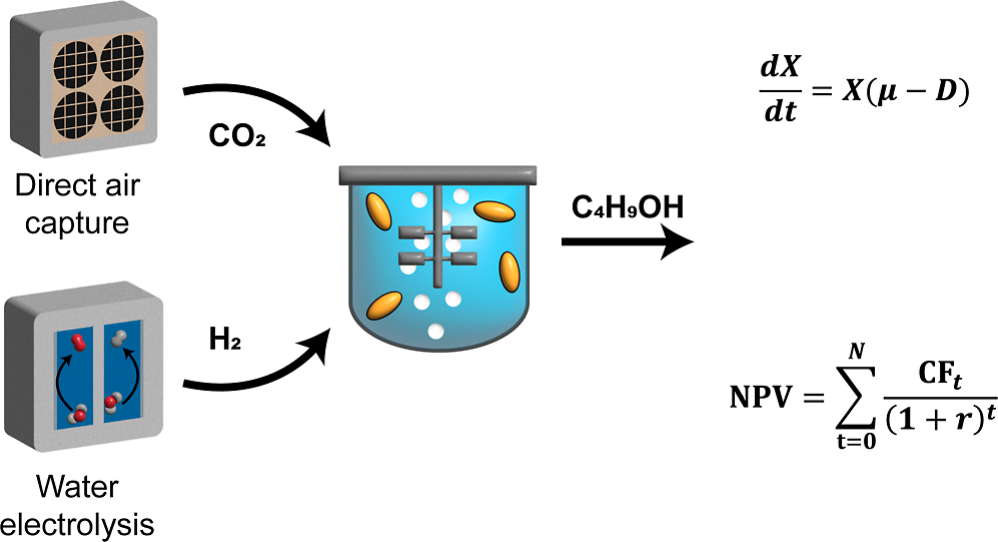

Electromicrobial production (EMP), where electrochemically generated substrates (e.g., H_2_) are used as energy sources for microbial processes, has garnered significant interest as a method of producing fuels and other value-added chemicals from CO_2_. Combining these processes with direct air capture (DAC) has the potential to enable a truly circular carbon economy. Here, we analyze the economics of a hypothetical system that combines adsorbent-based DAC with EMP to produce *n*-butanol, a potential replacement for fossil fuels. First-principles-based modeling is used to predict the performance of the DAC and bioprocess components. A process model is then developed to map material and energy flows, and a techno-economic assessment is performed to determine the minimum fuel selling price. Beyond assessing a specific set of conditions, this analytical framework provides a tool to reveal potential pathways toward the economic viability of this process. We show that an EMP system utilizing an engineered knallgas bacterium can achieve butanol production costs of <$6/gal ($1.58/L) if a set of optimistic assumptions can be realized.

## Abstract 2

Electromicrobial production is a next-generation biotechnology that could be used to produce carbon-neutral liquid fuels from CO_2_. While such systems have been proposed and demonstrated at the laboratory scale, we provide the first thorough techno-economic assessment of these processes.

## Introduction

Reliance on fossil fuels is a major contributing factor to anthropogenic climate change, given the large amount of carbon emitted during their production and use.^1^ Carbon capture and utilization seeks to replace fossil carbon feedstocks used in industrial production with CO_2_.^2^ Electrochemical,^3,4^ thermochemical,^5^ and biological^6^ processes have been developed to convert CO_2_ to fuels and other value-added products. Biological carbon utilization provides many advantages compared to traditional chemical and electrochemical processes, including operation at ambient temperature and pressure, innate catalyst regeneration, and high product selectivity. Feeding captured CO_2_ to photosynthetic organisms such as algae or cyanobacteria has been explored as a method of producing biofuels and other molecules of interest.^7,8^ Biological systems, however, are often slow relative to chemical processes, and the energy conversion efficiency in photosynthetic systems such as algal bioreactors is quite low (∼1.5–4.2%).^9,10^

Electrofuels, or e-fuels, on the other hand, use renewable electricity to convert CO_2_ to fuel. Many electrofuel strategies involve electrolysis of water to produce H_2_, which can then react with CO_2_ to form value-added products such as methane (Sabatier) or longer length hydrocarbons (Fischer–Tropsch).^11^ CO_2_ electrolysis systems have also been studied to electrochemically convert CO_2_ to value-added compounds.^12^ Recently, novel hybrid approaches referred to as electromicrobial production (EMP) systems, which combine electrochemical and biological processes to convert CO_2_ to value-added products, have been developed.^13^ Electrochemically produced substrates such as H_2_, CO, and HCOOH have been studied as microbial energy sources for bioproduction.^13^ Of these substrates, hydrogen gas can be produced through the most technologically mature and efficient processes (i.e., water electrolysis). H_2_ can be metabolized aerobically (by knallgas bacteria)^14,15^ and anaerobically (by acetogens and methanogens).^16,17^

While still in industrial infancy, direct air capture (DAC) processes relying on liquid solvents^18,19^ or solid-phase adsorbents^20,21^ can provide CO_2_ for carbon capture and utilization processes. Integrated DAC–EMP systems have the potential to convert electricity, water, and air into a seemingly endless array of products and can shift the paradigm from extractive petrochemical processing to a more circular carbon economy.^22^ Benchtop demonstrations of EMP systems producing biofuels, bioplastics, and other commodity chemicals have generated significant interest.^14,23,24^ Using EMP to convert CO_2_ to liquid fuels is of particular interest due to the immense carbon footprint of the transportation sector. While this approach is technically fascinating, the economics of such bioprocessing endeavors at industrial scales are not well understood due to a lack of thorough techno-economic analyses in the literature. While comprehensive analyses have studied the economics of conventional electrofuel processes (see, for example, the analysis by Sherwin),^25^ we are unaware of any similar analysis performed for the EMP of fuels.

A key factor limiting the ability to study the economics of EMP is the lack of demonstrations on a sufficient scale. Indeed, most examples of EMP systems in the literature have focused either on microbial engineering aspects or on the integration of electrochemical and biological systems at the laboratory scale, both of which provide limited data that can be used to directly assess the economics of hypothetical scaled-up systems. This gap has prompted work on modeling and analyzing EMP systems at various levels. Physical models of EMP, at both molecular and bioreactor levels, have been used to predict their hypothetical performance in terms of metrics such as productivity and energy conversion efficiency.^26,27^ Leger et al. devised a model to study the energy and land occupation footprints in the EMP of single-cell protein.^22^ We have recently developed a life cycle impact model to predict the environmental impacts of scaled-up EMP systems, demonstrating their promise from a sustainability perspective.^28^ Despite the significant contributions of each of these efforts, there is still a need for robust analyses that can bridge the gap between bench-scale demonstrations and understanding the economics of industrial-scale EMP.

Here, we study the economics of producing the biofuel *n*-butanol through a hypothetical scaled-up DAC–EMP process. As a drop-in replacement for gasoline, the cost targets for *n*-butanol production are well-established, and techno-economic analyses of *n*-butanol production through traditional bioprocesses allow for a clear basis of comparison.^29,30^ We study a hypothetical DAC–EMP process that contains a DAC module based on a solid adsorbent such as a metal–organic framework (MOF) and analyze two possible bioprocesses for converting CO_2_/H_2_ to *n*-butanol: a one-step process in which a knallgas bacterium directly converts CO_2_ to *n*-butanol and a two-step process in which an acetogen converts CO_2_/H_2_ first to acetate, with the acetate then converted to *n*-butanol by an acetotrophic microbe. We begin by developing physics-based model equations that predict the performance of the DAC and bioprocessing components. Process modeling then links these subcomponent models to predict the material and energy flows across the entire process. These flows are then translated into capital and operating costs of the process, and the minimum selling price of *n*-butanol is determined. As a result, the model can predict performance metrics required for economic viability and guide further research and development of these systems.

## Materials and Methods

### Resource Availability

MATLAB files used to implement the models described in this study are openly available in Open Science Framework (10.17605/OSF.IO/RG8TC).

### System Description

**Figure 1:**
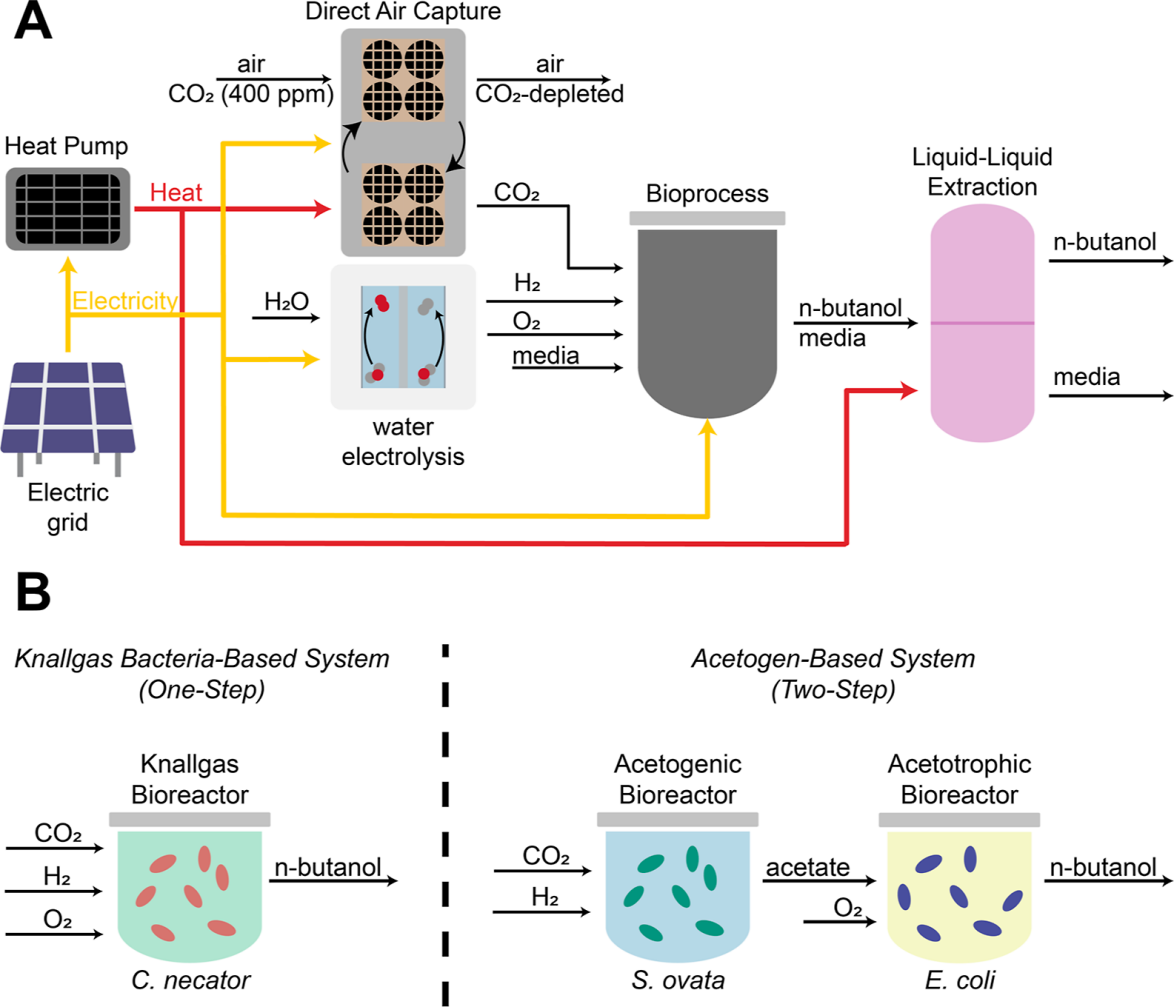
Schematic overview of DAC–EMP process. (A) Diagram of the DAC–EMP process showing the four major unit operations required for conversion of CO_2_, water, and electricity to *n*-butanol, as well as major material (black) and energy (electricity in yellow, heat in red) flows. (B) Diagram of the one-step bioprocess (left) relying on an engineered knallgas bacterium to convert CO_2_ to *n*-butanol and the two-step bioprocess (right) in which CO_2_ is first converted to acetate by an acetogen, and the acetate is subsequently upgraded to *n*-butanol by an engineered acetotrophic microbe.

Two hypothetical DAC–EMP processes for butanol production are examined here. Each of the processes is composed of four major subprocesses: a MOF-based DAC system, electrolysis to produce H_2_ from water, a biochemical process that converts H_2_ and CO_2_ to *n*-butanol, and a liquid–liquid extraction process that separates the *n*-butanol from the fermentation broth (Figure 1A). The DAC, electrolysis, and extraction subprocesses are the same in each of the hypothetical processes.

The DAC module is based on a temperature-vacuum swing adsorption process using a MOF as a solid sorbent in a catalytic monolith, similar to the process modeled by Sinha et al.^31^ Industrial fans are used to pass ambient air through a contactor containing the amine-functionalized MOF sorbent, onto which CO_2_ is selectively chemisorbed. Following the adsorption phase, CO_2_ is desorbed from the MOF, first by evacuating air from the contactor using a vacuum pump, and then using steam produced by a heat pump to bring the sorbent to the desorption temperature, liberating the captured CO_2_.

An electrolyzer is used to produce H_2_ for the process on-site, which serves as the energy source for carbon fixation. Two schemes for biochemically converting H_2_ and CO_2_ to butanol are considered (Figure 1B). In the first, H_2_, CO_2_, and O_2_ are fed to a bioreactor containing the knallgas bacterium *Cupriavidus necator*, engineered to produce n-butanol in a single step. The second option is a two-step system consisting of two bioreactors in tandem. One bioreactor containing the acetogen *Sporomusa ovata* converts H_2_ and CO_2_ to acetate (anaerobically), while a second bioreactor containing an acetotrophic microbe (e.g., *Escherichia coli*) converts the acetate to *n*-butanol (aerobically). All bioreactors are assumed to operate continuously in the liquid phase with constant bubbling of the substrate gases. Downstream of the bioprocessing step, butanol is first extracted from the medium into mesitylene and then distilled to separate it to the desired purity.

### Unit Operations and Process Modeling

The modeling and techno-economic approach for the DAC system borrows heavily from the approach taken by Sinha et al.^31^ Both the adsorption and desorption cycles of the DAC process are explicitly modeled. The DAC model equations are described in Note S1. These dynamic equations use various model parameters to calculate the productivity (CO_2_ captured per kg sorbent per hour), CO_2_ purity, and energy consumed by the DAC module.

The bioprocess model development follows the same basic methodology we described previously.^28^ The model equations for the knallgas bacteria-based process and the acetogen-based process are described in Note S2. Volumetric productivity (g BuOH L^–1^ h^–1^), butanol titer, and substrate consumption for each of the bioprocesses operating continuously at steady state are calculated from these model equations given a set of parameters and operating conditions.

The energy demand of the major unit operations (DAC blower, DAC vacuum pump, DAC heat pump, electrolyzer, and bioreactors) is described in Note S3. Mass and energy balances, combined with the results of the DAC, bioprocess, and separation models, are used to determine the material and energy demands for a desired production rate of *n*-butanol.

### Separations Modeling

CHEMCAD steady-state (https://www.chemstations.com) is used to simulate the liquid–liquid extraction and distillation processes used to separate butanol from the fermentation broth. The UNIFAC LLE model was used to predict thermodynamic parameters, with all other thermodynamic settings left at the CHEMCAD default values. Water, mesitylene, and *n*-butanol are the only components considered. The flow rate of mesitylene into the extractor relative to the flow of the fermentation medium is set such that 99% of the generated *n*-butanol is extracted. The first distillation column is defined to remove water such that the remaining weight fraction is 0.5% of that of butanol, and the second distillation column is defined to separate the maximum amount of *n*-butanol while the mesitylene weight fraction in the product stream remains less than 0.5% (final butanol purity >99%). The mesitylene from the bottom fraction of the second distillation column is then recycled for further extraction of butanol. Heat exchangers transfer heat from this hot mesitylene stream to the mesitylene-rich fraction leading to the distillation columns, recycling some of the heat used in the separation process (a minimum temperature difference of 10 °C is assumed). The flow rate of mesitylene, consumption of mesitylene, product recovery fraction, energy demands of the distillation columns, and sizes of distillation columns calculated here are then used in the broader process model and techno-economic analysis.

### Techno-Economic Modeling

We assume that the process will run 24 h a day with an annual uptime of 330 days. We use a constant dollar approach to cash flow modeling,^32^ with 2022 as the reference year. Therefore, we assume that butanol production begins in 2022, all equipment is costed in 2022 dollars, and the material/labor costs are based on 2022 prices for the duration of the project. The process is assumed to produce 40 million gallons of butanol per year (120,000 t/y), comparable in scale to previously reported techno-economic assessments for lignocellulosic ethanol plants.^33^

Equipment sizes and the number of each equipment item required are determined from the process model described in the preceding section. The costs of equipment obtained through established correlations and literature searches are adjusted for inflation to 2022 dollars with the Chemical Engineering Plant Cost Index used as the cost index. The correlations and installation factors used to determine the cost of the major pieces of equipment used in this process are listed in Table S2, Supporting Information. Heuristics are used to estimate the total capital investment from the installed equipment cost, following the assumptions made in reports published by the National Renewable Energy Laboratory describing a plant for lignocellulosic biofuel production (see Table S4, Supporting Information).^33,34^

Electricity production is assumed to take place outside of the system boundary, and the analysis assumes that electricity can be purchased at a fixed price ($0.05/kW h in the base-case analysis, based on the levelized cost of solar electricity in a medium resource area).^35^ Unit costs of other materials/utilities used in this process are given in Table S3, which, along with the process model, are used to calculate the variable operating cost (VOC) of the process. The method of calculating other operating cost contributions is detailed in Table S5. A discounted cash flow rate of return (DCFROR) analysis is performed to determine the minimum selling price for *n*-butanol produced in the hypothetical plant described here. Parameters used in the DCFROR analysis, including discount rate, depreciation method, plant life, construction period, and equity financing, are summarized in Table S6.

## Results and Discussion

### Modeled Performance of DAC System

We began by modeling the DAC component of the DAC–EMP process. The model equations for the DAC system described in Note S1 are indifferent to the exact type of adsorbent used. However, for the analysis here, we use parameters based on the MOF mmen-Mg_2_(dobpdc), first synthesized by McDonald et al.^36^ Isotherm data (see Note S4), sorbent capacity, density, heat capacity, adsorption kinetics, and adsorption thermodynamics used in the model here match those reported for mmen-Mg_2_(dobpdc). In addition to the physical parameters, the behavior of the DAC module is dependent on the geometry of the contactor and other operating conditions. Base-case parameters and operating conditions are listed in Table S1.

**Figure 2:**
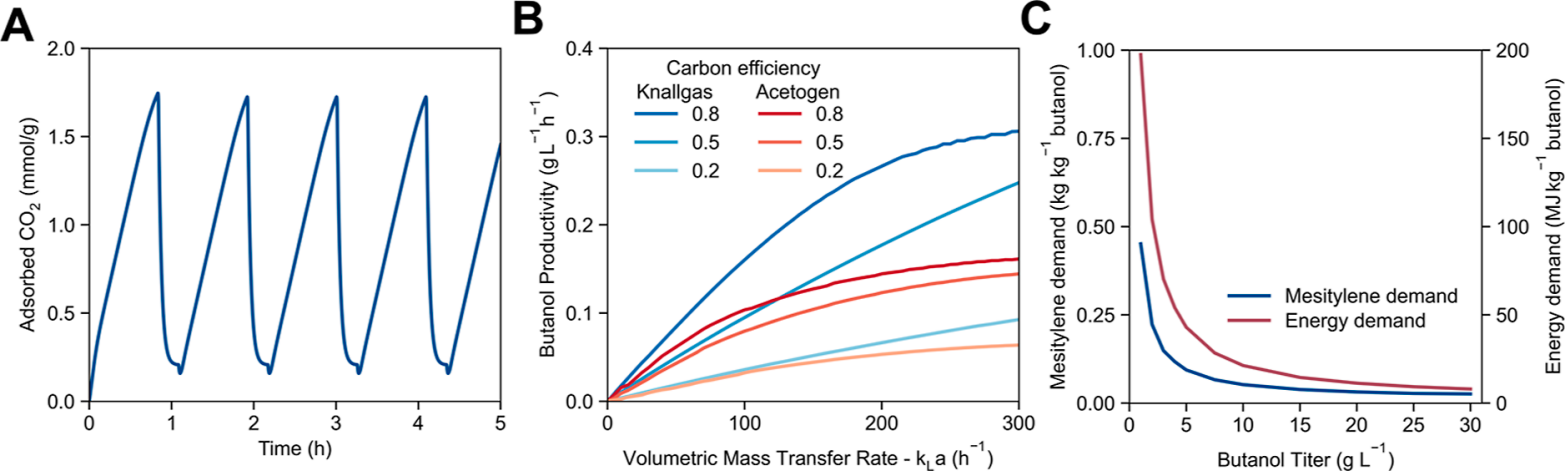
Modeled performance of the subprocesses in the DAC–EMP system. (A) Representative adsorption and desorption cycles of the DAC process modeled at base-case parameters and operating conditions. (B) Modeled butanol productivity as a function of volumetric gas–liquid mass-transfer rate for H_2_ for both the knallgas bacteria-based (1-stage) and acetogenic bacteria-based (2-stage) systems for butanol production at a variety of assumed carbon selectivities (fraction of fixed carbon embodied in *n*-butanol rather than in biomass). (C) Effect of the *n*-butanol titer from the bioprocess on the mesitylene (blue) and energy (red) required to purify *n*-butanol to 99% purity.

Simulated breakthrough curves were obtained first (Figure S2, Supporting Information). Under base-case operating conditions, the rate of adsorption slows after ∼45 min, with the sorbent reaching its maximum capacity after ∼75 min. This breakthrough curve is similar to the one modeled by Sinha et al., who also modeled the performance of MOF mmen-Mg_2_(dobpdc).^31^ The final conditions of the adsorption model can serve as the initial conditions of the desorption model described in Note S1. In this model, the rate of heating the contactor is quite fast, and the dynamics of the desorption process are controlled by the rate at which CO_2_ desorbs from the adsorbent. Once the desorption dynamics are modeled, those final conditions can serve as the initial conditions for the adsorption model, and DAC cycles can be simulated (Figure 2A).

The difference in the adsorbed CO_2_ concentration at the end of the adsorption process and at the end of the desorption process is taken to be the amount of captured CO_2_ per cycle (Δ*q*_cycle_). The productivity, in mol of CO_2_ (kg of adsorbent)^−1^ h^–1^, can then be calculated by dividing this value by the total cycle time, including the adsorption step, desorption step (15 min), and an assumed dead time of 3 min per cycle. In practice, the sorbent would not become saturated each cycle, as the rate of CO_2_ adsorption decreases when nearing its maximum capacity; therefore, the productivity can be maximized by varying the adsorption time. For the base-case parameters and operating conditions, a maximum productivity of 1.34 mol kg^–1^ h^–1^ occurs at an adsorption time of 50 min, which corresponds to a (Δ*q*_cycle_) of 1.52 mol kg^–1^ (see Note S5).

### Modeled *n*-Butanol Productivity of Knallgas Bacteria- and Acetogen-Based EMP Systems

CO_2_ captured by DAC is then converted to *n*-butanol by a bioprocessing step in the hypothetical process evaluated. The model equations for both the knallgas bacteria- and acetogen-based systems are implemented using the base-case parameters described in Table S1. It is assumed that the gaseous substrates are fed in the stoichiometric proportions that they are consumed in. While the equations described are dynamic, the bioreactors (all modeled as chemostats) operating at a given dilution rate will reach a steady state, and those steady-state conditions are used to evaluate the system. There will be a dilution rate that maximizes the productivity of each bioreactor. For the first bioreactor in the acetogen-based system, the productivity of acetate generation is optimized, while butanol productivity is optimized in the knallgas bacteria-based system and in the second bioreactor in the acetogen-based system (see Note S6). The productivity of each of the bioprocesses depicted in Figure 2B assumes that the bioreactors are operating at the dilution rate that maximizes the productivity of the bioreactor(s) under a given set of conditions.

The butanol productivity in each system naturally depends on the carbon selectivity (Figure 2B), defined here as the fraction of carbon in the form of *n*-butanol compared to the carbon in all products (butanol and biomass) generated in the bioreactor (note: this carbon selectivity is equal to 1 – Φ, as defined by the model equations in the Supporting Information). Carbon selectivities as high as 0.8 have not yet been achieved in the conversion of CO_2_/H_2_ or acetate to butanol, although they have been achieved for other substrates (e.g., glycerol, glucose).^37,38^ While thermodynamically possible, it is likely that achieving yields this high will require substantial metabolic engineering. Metabolic engineering strategies to manage carbon flux toward desired products are well established in the literature,^39^ and genetic tools now exist for hydrogenotrophic strains (although we note that, to date, genetic toolkits for hydrogenotrophs such as *C. necator* are underdeveloped compared to those for *E. coli*).^40,41^ A carbon selectivity of 0.8 will be assumed for the rest of this analysis to demonstrate how the system would perform if such a target is met.

In the acetate-mediated bioprocess, the whole-system productivity increases with increasing *k*_L_*a*, yet it quickly begins to plateau. This occurs as other factors limit productivity, most notably salt toxicity. Substantial quantities of NaOH are required to balance the pH during acetogenesis, and because one mole of NaOH is added per mole of acetate produced, salt toxicity limits the titer of acetate. This limits the productivity of the acetogenic bioreactor as well as that of the downstream acetotrophic bioreactor. Assuming a carbon selectivity of 80%, the overall productivity of the acetogen-based bioprocess is predicted to reach 0.16 g of BuOH L^–1^ h^–1^ as the *k*_L_*a* reaches 300 h^–1^ (Figure 2B).

The productivity of the knallgas bacteria-based system, meanwhile, is predicted to reach 0.31 g L^–1^ h^–1^ under these same conditions (Figure 2B). In both systems, the rate of gas–liquid mass transfer, hindered by the low solubility of hydrogen gas in water, places limits on the productivity of the bioprocess. We note, however, that as the *k*_L_*a* value continues to increase, the system instead becomes limited by the effects of butanol toxicity, and therefore increases in the *k*_L_*a* only provide marginal increases to productivity. The productivity in the one-stage system is highly dependent on the tolerance of the knallgas bacteria to *n*-butanol (see Note S7). The modeled titer of the acetogen-based system remains lower (∼5 g L^–1^), and therefore, issues regarding butanol toxicity are unlikely to emerge under these conditions. Improvements to the butanol tolerance of knallgas bacteria beyond the 10 g L^–1^ limit assumed here are an important area of further research to improve the productivity of this system.

The necessity of two reactors in the acetate-mediated system causes the total reactor volume to be higher than that of the knallgas bacteria-based system. Therefore, even if equivalent rates of gas–liquid mass transport are achieved in both systems, the overall productivity of the two-stage system will be lower. Moreover, salt toxicity is not encountered in the knallgas bacteria-based system. Our previous modeling work has also shown that the knallgas bacteria-based system will have a higher productivity than an acetogen-based system when producing biomass or hypothetical products such as industrial enzymes or lactic acid, for similar reasons.^28^

Both bioprocess options were then modeled to examine the effect of gas recycling (see Note S8). According to the model, the gases can be substantially recycled (99% of vented gas is recycled) without a decrease in productivity. After this point, further recycling will lead to accumulation of impurities (namely N_2_ gas from the DAC system), leading to a rapid decrease in the productivity of the system. For further studies, it is assumed that 99% of the vented gas is recycled.

### Material and Energy Requirements for Separation of *n*-Butanol from the Fermentation Broth

The chemical process modeling software CHEMCAD was used to simulate the process of purifying *n*-butanol from the effluent of the bioprocessing step, which requires extraction of *n*-butanol by mesitylene and two distillation steps, first to remove any extracted water and then to purify the *n*-butanol. For a given *n*-butanol titer and purity requirement (>99%), the simulation could predict the flow rate of mesitylene required, the amount of mesitylene consumed by the process, the energy demands of the distillation columns, and the fractional recovery of *n*-butanol. The simulation is also used to size the extractor, distillation columns, and heat exchangers used in the process, which will be factored in during the capital cost calculations. The mesitylene demand and energy requirements of the distillation columns are nearly inversely proportional to the starting *n*-butanol titer (Figure 2C).

### Process Modeling and Base-Case Techno-Economics

Under base-case assumptions and operating conditions (see Table S1), the knallgas bioreactor is predicted to achieve a productivity of 0.295 g L^–1^ h^–1^, a yield of 1.57 g butanol/g H_2_, and a butanol titer of 10.0 g L^–1^. The first bioreactor in the acetogen-based system is predicted to achieve an acetate productivity of 1.44 g L^–1^ h^–1^ and an acetate titer of 22.7 g L^–1^, while the second bioreactor operates with a butanol productivity of 0.296 g L^–1^ h^–1^ and a butanol titer of 5.4 g L^–1^. Considering both bioreactors, the acetogen-based system realizes an overall H_2_-to-butanol yield of 1.62 g/g and an overall productivity of 0.155 g L^–1^ h^–1^. Taking these results, along with the models for the DAC and separations components, material and energy demands for the chosen production rate of butanol (40 MM gal/y) can be calculated. Process demands assuming base-case parameters and operating conditions are summarized in Table 1 (for detailed process flow diagrams and material flows, see Figures S6, S7, Tables S10 and S11).

**Table 1:** Material and Energy Flows in DAC–EMP Process for 40 MM gal/y (120,000 t/y) Butanol Production

process component	process demand—knallgas	process demand—acetogen	cost per gallon BuOH—knallgas	cost per gallon BuOH—acetogen
energy demands				
DAC blower	6.3 MW	13.7 MW	$0.06	$0.14
DAC vacuum pump	4.8 MW	10.5 MW	$0.05	$0.10
DAC heat pump	28.5 MW	62.2 MW	$0.28	$0.62
electrolyzer	509.4 MW	494.3 MW	$5.04	$4.89
bioreactor energy	12.6 MW	32.3 MW	$0.12	$0.32
separations	37.9 MW	68.6 MW	$0.38	$0.68
**total electricity**	**599.5 MW**	**681.6 MW**	**$5.93**	**$6.75**
material demands				
sorbent^a^	0.05 t/h	0.11 t/h	$0.65	$1.42
monolithic support^a^	0.01 t/h	0.03 t/h	$0.01	$0.03
nitrogen gas	0.5 t/h	1.1 t/h	$0.07	$0.16
electrolysis water	92.4 t/h	89.7 t/h	$0.02	$0.02
ammonia	0.9 t/h	1.4 t/h	$0.21	$0.33
phosphoric acid	0.4 t/h	0.7 t/h	$0.11	$0.17
magnesium sulfate	0.4 t/h	0.7 t/h	$0.03	$0.05
sodium hydroxide	0 t/h	42.9 t/h	$0.00	$5.35
sulfuric acid	0 t/h	51.6 t/h	$0.00	$2.65
mesitylene	0.9 t/h	1.5 t/h	$0.70	$1.18
waste disposal and treatment^b^			$0.53	$1.37
**total material**			**$2.34**	**$12.74**
**process total**			**$8.28**	**$19.49**

a Both the DAC sorbent and monolithic support are considered as material demands, despite their long lifetime relative to other materials used in the process. The process demand, in t/h, is the amount of sorbent required to maintain the given butanol production rate divided by the lifetime of the sorbent (*t*_DAC_, 2 years in the base case).

b Description of the various costs associated with waste disposal and wastewater treatment is provided in Note S9.

While various parts of the process require energy in the form of both electricity and heat (for example, the DAC process requires heat for catalyst regeneration and electricity for operating the fans and vacuum pumps), we assume that heat pumps are employed. By the employment of industrial heat pumps, all energy required by the process can be delivered by electricity. As EMP will only be practical if renewable electricity is cheap and abundant,^28^ electrifying the entire process would be beneficial. Moreover, making this assumption allows a direct comparison of the energy demands of all unit operations, regardless of whether electricity or heat is required. The power demand of the entire process to meet the desired production rate of 40 MM gal/year is over half a gigawatt, slightly lower than the output of the largest photovoltaic power station in the United States at the time of writing.

Hydrogen production is the most energetically expensive component of the process for both bioprocess options. This is conceptually unsurprising, as hydrogen is the energy carrier driving the conversion of CO_2_ to *n*-butanol. Although separating CO_2_ from atmospheric concentrations is energetically costly, the electrolysis component of the process requires about an order of magnitude more power than the DAC. To produce the quantity of CO_2_ required to run the knallgas bacteria-based system, 385,000 tons per year must be captured through the DAC component (roughly twice this value is needed for the acetogen-based process). This is much larger than any DAC plant constructed to date, although a 500,000 t/y DAC plant is (at the time of writing) under construction by 1PointFive.^42^

In the knallgas bacteria-based system, 119 kW h of electricity is consumed per gallon (31.2 kW h/L) of *n*-butanol produced, which, when considering the energy content of the produced fuel, works out to 1.07 kW h consumed per MJ fuel. This corresponds to a whole-process energy conversion efficiency of 25.8%. When considering only the electricity required to run the electrolyzer and discounting the hydrogen that is used to produce biomass rather than *n*-butanol, the energy efficiency increases to 42.4%, which represents the upper limit of electricity-to-fuel efficiency predicted by our model. This is in close agreement with the energy efficiency of the CO_2_-to-butanol process modeled by Salimijazi et al. (44.6%).^26^

Claassens et al. placed an upper energy efficiency limit for EMP systems using knallgas bacteria at 35% (or 28% when factoring in the efficiency of hydrogen production). Our modeled energy efficiency exceeds this value for two possible reasons. First, Claassens et al. based their analysis on empirical knallgas bacteria growth data, in which practical energy losses that are not captured by our theoretical model likely occur. Second, and more important, they performed their analysis based on the production of biomass, which includes myriad complex biochemical reactions that will likely lead to greater energy inefficiency compared to butanol production (indeed, our previous analysis of an EMP process producing only biomass predicted an energy efficiency of only 23%).^28^ Compared to experimental EMP systems, Liu et al. reported a maximum electricity-to-fuel efficiency of 27% when using their “Bionic Leaf” system to convert CO_2_ and electrolytically generated H_2_ to C_4_ and C_5_ alcohols.^23^ According to our model, this indicates that the Bionic Leaf system was able to achieve energy conversion efficiencies >60% of the theoretical maximum. Our model, in addition to predicting the hypothetical potential of EMP systems, can be used to assess the performance of actual systems developed at the benchtop scale.

The VOC of the process can be calculated by multiplying the energy and material demands by their unit costs (see Table S3). Under base-case assumptions and operating conditions, the VOC values of the knallgas bacteria- and acetogen-based systems are $8.28 and $19.49 per gallon ($2.18 and $5.13 per liter), respectively. A major takeaway from this initial analysis of the VOC is the relative advantage of the knallgas bacteria-based system compared to the acetogen-based system. For nearly every process demand analyzed, the acetogen-based process requires more material/energy than the knallgas bacteria-based process. The only exceptions are the demands related to hydrogen production. The Wood–Ljungdahl pathway is a more energy-efficient form of carbon fixation than the Calvin cycle. However, this advantage is almost completely lost due to the energy inefficiency in converting acetate to butanol. Therefore, the acetogen-based system only uses slightly less hydrogen than the knallgas bacteria-based system. However, all other demands are higher in the acetogen-based system, mostly due to three issues: the higher demand for CO_2_ (as there is significant CO_2_ loss in the second bioreactor), the lower butanol titers, and the necessity for pH control.

Of these disadvantages, the most striking is the large contribution of sodium hydroxide and sulfuric acid used to control the pH in the two-stage system. These pH control materials alone account for around 40% of the VOC ($8/gal), more than all of the electricity used. The necessity of pH control in both the acetate-producing and acetate-consuming components of the bioprocess is a significant downside of this particular EMP scheme. Conversion of H_2_/CO_2_/O_2_ to butanol and biomass in the knallgas bacteria-based system involves no net generation or consumption of protons, and therefore pH control is not a major material cost in that system. Therefore, unless the need for pH control can be obviated in the two-microbe system, the knallgas bacterium-based system has a clear economic advantage.

### Capital Costs and Base-Case Minimum Fuel Selling Price

Based on the material and energy flows, the major pieces of process equipment may be sized, and their cost may be estimated based on established correlations and other literature data (see Table S7). The largest component of the installed equipment cost comes from the bioreactors ($555 MM for the acetogen-based system and $296 MM for the knallgas bacteria-based system) and the electrolyzer ($467 MM for the acetogen-based system and $481 MM for the knallgas bacteria-based system). Factoring in installed equipment costs and other capital expenses (see Table S4), the total capital investment is $2.6 billion for the acetogen-based system and $1.9 billion for the knallgas bacteria-based system. For reference, a techno-economic analysis for lignocellulosic ethanol production operating at a capacity of 61 MM gal/y (equivalent in energy content to 47 MM gal/y of *n*-butanol) calculated a total capital investment of $423 MM in 2007$ (or $641 MM when inflated to 2022$).^33^ A techno-economic analysis for butanol produced from wheat straw at a capacity of 147,000 tons butanol per year (48 MM gal/y) estimated a capital investment of $296 MM when converted to 2022$.^43^

Two main factors cause the discrepancy between this analysis and those for other biofuel processes: the contribution of the electrolyzer and the larger contribution of bioreactors in EMP. The procurement of the microbial feedstocks in conventional bioprocesses (in the form of either sugars or lignocellulose) does not add significantly to the capital cost of those processes; in EMP processes, however, large-scale electrolyzers (and carbon capture equipment) are needed to generate the substrates. The large contribution of the bioreactors to the capital cost of the modeled EMP processes stems from the relatively low volumetric productivities compared with other processes. We predict productivities as high as ∼0.3 g L^–1^ h^–1^ in the base-case scenario, compared to 1.7 g L^–1^ h^–1^ in ethanol fermentation processes.^34^ The productivities of the gas fermentation systems analyzed here are primarily limited by gas–liquid mass-transfer rates (due to the low solubility of H_2_), an issue not encountered in traditional ethanol and butanol fermentation processes. Therefore, to produce equivalent amounts of fuel, EMP processes will need more/larger fermenters. While the low solubility of hydrogen has often been considered a major challenge,^13,26^ our model is able to quantify the impact that this physical challenge imposes on the bioprocess productivity and consequently on the economics of the process.

A DCFROR analysis can then be performed to determine the minimum butanol selling price. In the base-case scenario, given a discount rate of 10%, the minimum selling price is $29.27/gal ($7.70/L) for the acetogen-based system and $15.33/gal ($4.03/L) for the knallgas bacteria-based system (Table S8). Both prices are significantly higher than those of comparable liquid transportation fuels. Retail gasoline prices averaged $3.97/gal ($1.05/L) in 2022,^44^ while corn ethanol fuel in the United States cost $4.13 per gallon ($1.09/L) of gasoline equivalent in October of 2022.^45^ We note that although *n*-butanol is often discussed as a “drop-in” replacement to gasoline, the energy content of butanol fuel is ∼14% lower than that of gasoline, which should be taken into consideration when comparing prices.^46^ Techno-economic assessments of biobutanol produced from lignocellulose or food waste placed a selling price around $4/gal ($1.05/L).^29,43^ The price difference between EMP-derived biofuels in this base-case analysis and conventional biofuels stems from the large difference in capital costs (described above) and the cost of the substrates in each system.

While this analysis specifically models the production of *n*-butanol, the economics of EMP processes, in general, can be inferred. For example, producing other fuels, such as jet fuel, in an EMP system would be beneficial. Recent analyses suggest that a model jet fuel blend could be produced by knallgas bacteria with similar energy efficiencies as *n*-butanol, assuming sufficient genetic engineering is done.^26,47^ Therefore, considering the costs per fuel content allows for a basis for comparison between different fuel types. In the base-case scenario, fuel can be produced by the one-step and two-step processes for $0.14 and $0.26 per MJ, respectively. At the end of 2022, jet fuel in the US averaged $3.32/gal, or $0.025/MJ, although sustainable aviation fuels are generally more than twice as expensive as conventional jet fuel.^44,48^ Broadening the comparison to other products beyond fuels, the production costs for the two systems per unit mass ($9.51 and $4.98 per kg butanol) are significantly higher than the often-cited ∼$1/kg target for biologically produced commodity chemicals.^49,50^ Clearly, improvements to the EMP systems described here must be made in order to attain economic viability as a general bioprocessing strategy.

### Pathway to Economic Feasibility for DAC–EMP Systems

**Figure 3:**
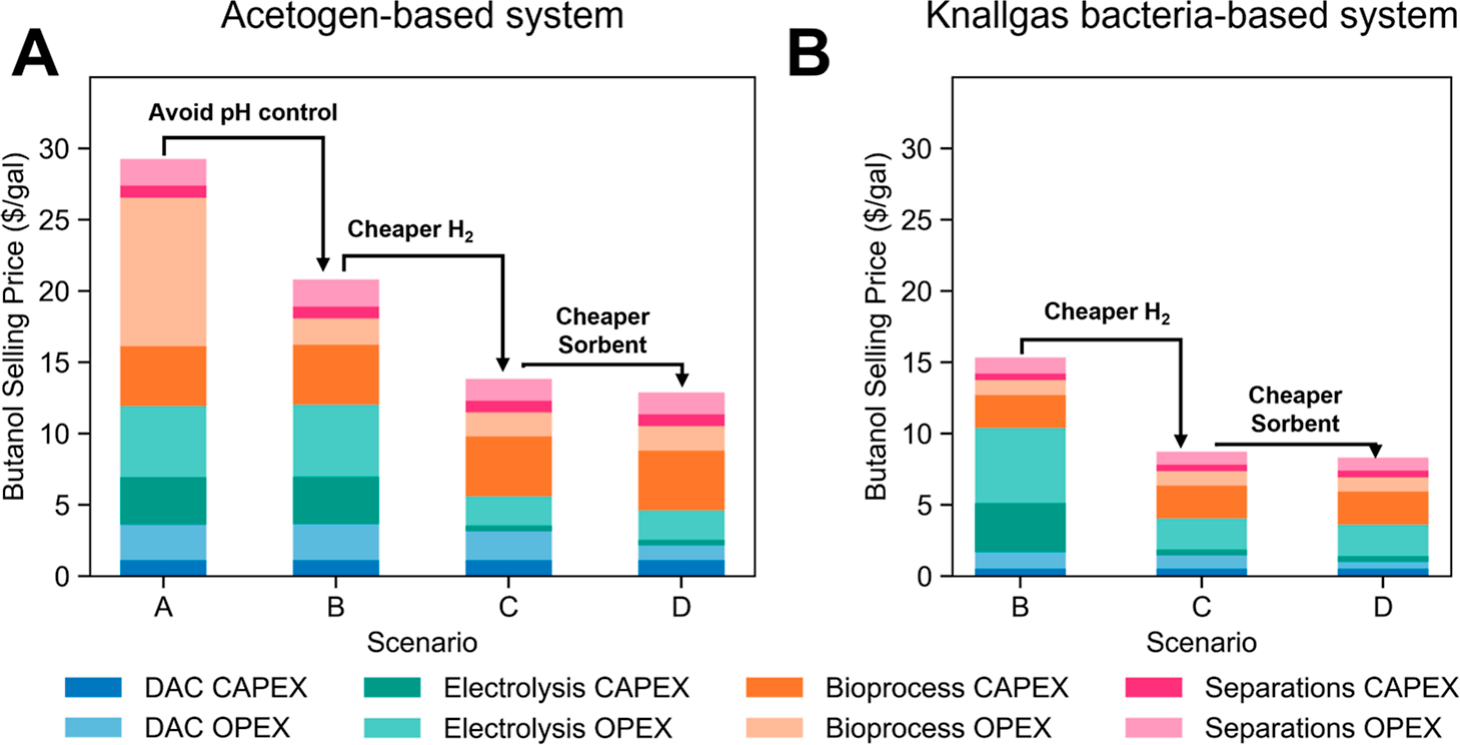
Potential pathways for improving economics of *n*-butanol production. Demonstration of how different scenarios will progressively lead to decreased butanol costs in the (A) acetogen- and (B) knallgas bacteria-based systems, starting from the base-case scenario. Scenario B describes a scenario in which pH control is no longer necessary (only applies to acetogen-based system). Scenario C describes a scenario in which the electrolysis capital cost falls to $100/kW and the electricity price falls to $0.02/kW h, consistent with the 2030 goals of the U.S. Department of Energy’s Hydrogen Shot Initiative. Scenario D describes a process with the DAC sorbent decreasing in cost by two-thirds compared to the base-case cost.

The base-case cost of butanol described in the previous section is based on many assumptions that may change for a variety of reasons. Prices of individual materials may rise or fall, better-performing materials and biocatalysts may be developed, and in many cases, the value of a given parameter is subject to significant uncertainty. Taken together, the models of the individual unit operations, the process-level mass and energy balances, and the techno-economic model can evaluate the impact of various parameters on the overall economics of the DAC–EMP process described here. Successive scenarios that improve the process economics can be evaluated until the minimum selling price of butanol is within the ballpark of being competitive with petroleum-based fuel (Figure 3).

The acetogen-based system will be examined first, with a minimum butanol selling price of $29.27/gal ($7.70/L) in the base-case scenario. From here, other scenarios could be considered. The first modification assumes that the need for pH control can be removed. In theory, this could be achieved if microbial strains that tolerate pH extremes could be used in this process, although this may be challenging, given the large amount of acetate produced. As an alternative strategy, if the two steps can be combined in a single reactor and acetate production and consumption occur at similar rates, the environment can remain neutral with little or no addition of acid or base. This would require an acetogen that is not strictly anaerobic, as acetate consumption requires oxygen. Adaptive laboratory evolution has been used to develop an oxygen-tolerant strain of *S. ovata*,^51^ indicating that this may be a promising strategy. Eliminating the need for pH control decreases the minimum selling price of butanol fuel by over $8/gal.

The next change examines the impact of the expected decline in the hydrogen production costs. The economic argument for hydrogen-mediated EMP rests on hydrogen being a cheaper substrate than sugars, such as glucose. Meeting the goal of the US DOE’s Hydrogen Shot Initiative ($1/kg H_2_) will require electrolyzer capital costs to decline from $900/kW to $100/kW, along with electricity becoming available at $0.02/kW h.^52^ These assumptions reduce the butanol cost by $7/gal. At this point, the DAC cost becomes a meaningful contributor to the process’s economics. The next scenario considers a 3-fold reduction in the synthesis cost of the adsorbent. This lowers the cost of DAC from $180/ton to $105/ton, which would be in line with optimistic projections of DAC systems operating at a large scale,^19^ decreasing the butanol cost by ∼$1, to $12.88/gal ($3.39/L).

The knallgas variation of the DAC–EMP process can then be considered. In the base-case scenario, the selling price of *n*-butanol is $15.33/gal ($4.03/L). The knallgas system does not require substantial pH control. Therefore, the first modification to remove the cost of pH control is unnecessary for this system. Cheaper H_2_, as described before, reduces the butanol selling price to $8.73/gal ($2.30/L), while decreasing the cost of the DAC sorbent further reduces the butanol price to $8.30 ($2.18/L). This exercise highlights the utility of this framework to evaluate several different scenarios to understand possible paths to the economic viability of a DAC–EMP system, which can aid in driving research directions toward addressing the roadblocks identified.

### Biochemical Engineering Targets for Electromicrobial Production of *n*-Butanol

As demonstrated in the previous section, several exogenous factors contribute significantly to the economic viability of biofuel production. Assuming that these requisite conditions (reduced H_2_ and CO_2_ costs) are met, the performance of the bioprocesses themselves, specifically in terms of volumetric productivity and butanol titer, becomes important. Therefore, we analyzed the effect that changes in productivity and titer have on the minimum fuel selling price in order to determine the conditions required to lower the butanol selling price to $6/gal ($1.58/L). While $6/gal is still roughly 50% higher than the average price of gasoline in the US in 2022, regional and temporal variations in gasoline prices could allow EMP to be cost-competitive at that price (indeed, gasoline prices approached $6/gal on the West Coast of the US during this period).^45^ Moreover, the implementation of public policy measures (e.g., carbon taxes, carbon utilization credits, and hydrogen production credits) could allow EMP to be cost-competitive nationally, although such calculations are beyond the scope of this analysis.

**Figure 4:**
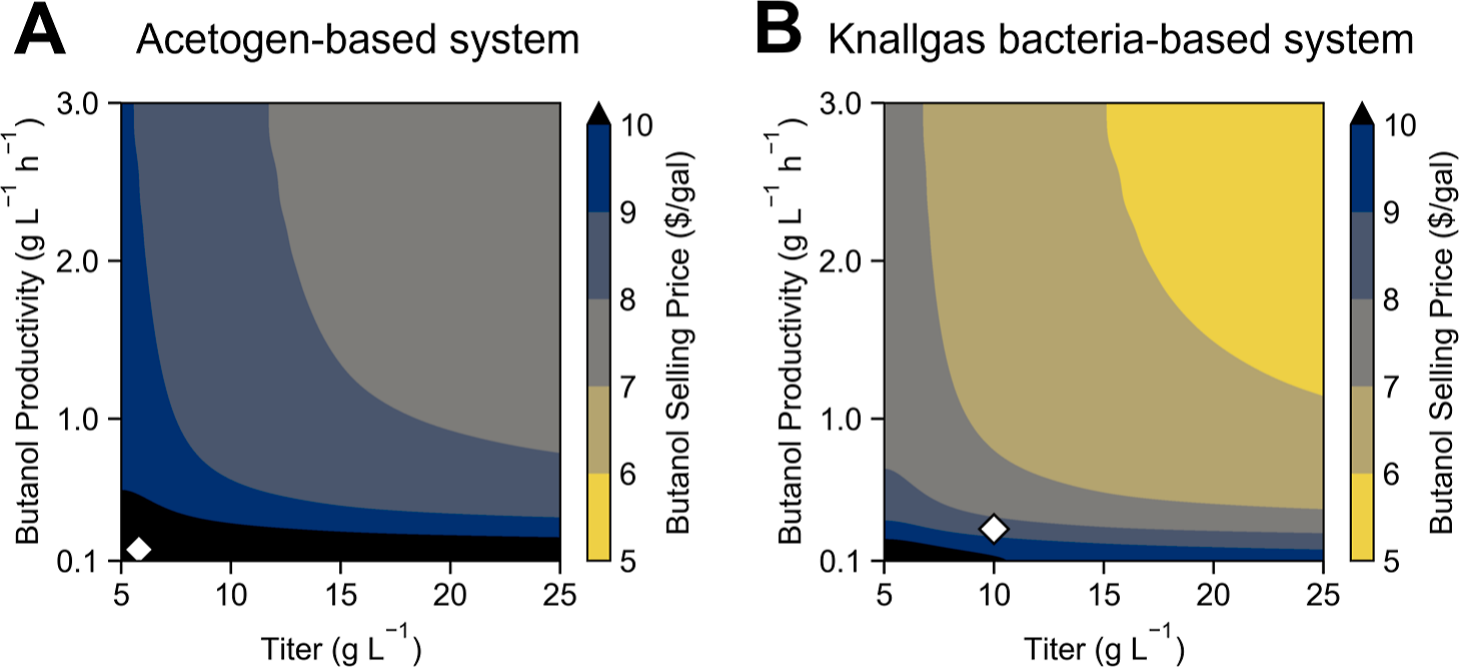
Effect of biochemical engineering metrics on the economics of DAC–EMP biofuel processes. Independent influence of the volumetric productivity and titer on the selling price of butanol per gallon for the two-stage (A) and one-stage (B) DAC–EMP system. White diamonds represent the titer and productivity modeled by each system under base-case assumptions.

Values of the productivity and titer of each system were independently varied, with all other model parameters held at the same values as in scenario D described in Figure 3, and the butanol price was recalculated (Figure 4). Naturally, higher titers and productivities lead to lower butanol selling prices. As productivity increases, the capital cost of the bioreactor per kilogram of butanol is reduced. Meanwhile, as the butanol titer increases, the material/energy demand and capital costs associated with the separation of butanol will decrease. For the knallgas bacteria-based system, titers above ∼15 g L^–1^ and productivities above ∼1.5 g L^–1^ h^–1^ are required to achieve a butanol selling price under $6/gal (Figure 4B). These targets are higher than those predicted by the model described earlier (∼0.3 g L^–1^ h^–1^ productivity with 10 g L^–1^ titer), suggesting that a simple chemostat may not reach the productivity and titer required for economic viability, and novel bioreactor strategies may be necessary. In the acetogen-based system, even with titers above 25 g L^–1^ and productivities above 3 g L^–1^ h^–1^, butanol selling prices remain above $7/gal (Figure 4A). The major reason for this discrepancy is the higher CO_2_ demand in the acetogen-based system, as well as the higher demand for nutrients (ammonia, phosphate, etc.) when culturing bacteria in two separate bioreactors.

### Parameter Sensitivity Analysis

**Figure 5:**
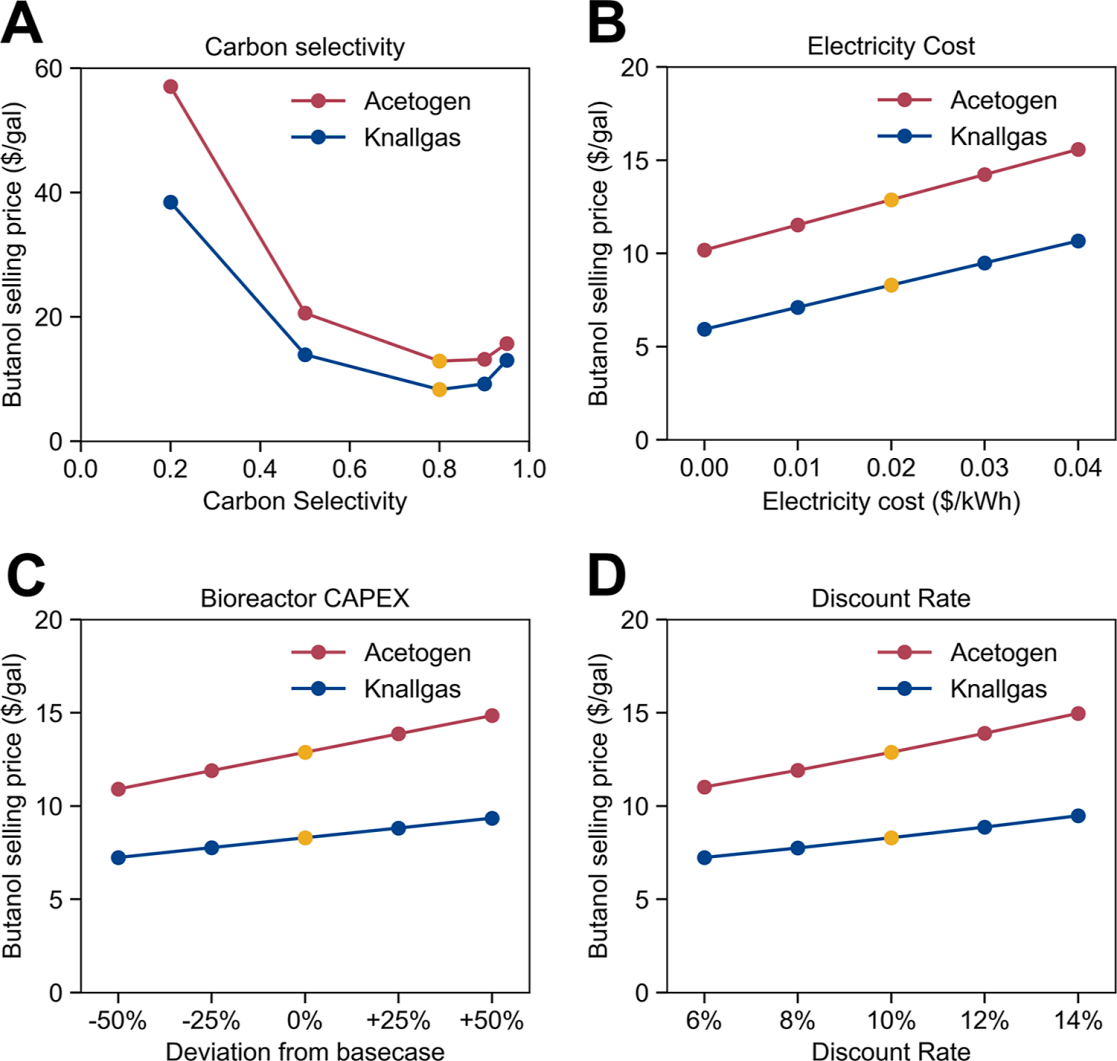
Sensitivity analysis of select parameters. (A) Effect of carbon selectivity (moles of carbon embodied in butanol compared to total moles in both butanol and biomass) on the butanol selling price. (B) Effect of electricity cost on the butanol selling price. (C) Effect of bioreactor cost (represented as percent deviations from the base value) on the butanol selling price. (D) Effect of the discount rate on the butanol selling price. Yellow markers denote the base-case parameter values in the optimistic scenario described in Figure 3. Effects of other parameters can be found in Table S9.

As demonstrated in previous sections, the results of this techno-economic analysis are sensitive to the values of various input parameters, which, given the inherent uncertainty of many of these parameters, limits the certainty of the final butanol selling price. Bioreactor model parameters, process model parameters, material costs, equipment costs, and financial assumptions directly affect the economics of the process. To study these effects, single-parameter sensitivity analyses were performed to understand which parameters most significantly impact the final economic results. Parameters were varied according to a range deemed reasonable for their specific value, with the justification for that range documented in Note S10. In this analysis, scenario D as described by Figure 3 (the most optimistic scenario) is used as the baseline for comparison. A sample of interesting results is highlighted in Figure 5, while comprehensive results of all 38 parameters tested are listed in Table S9.

Of the bioreactor model parameters, the carbon selectivity most significantly impacts the economics of butanol production. In general, as the carbon selectivity increases, the cost of butanol production decreases as lower material demands (most notably, H_2_ and CO_2_) are required. However, as the carbon selectivity increases above 0.8, the cost of butanol counterintuitively begins to increase. This interesting result stems from the fact that chemostat bioreactors are modeled. As selectivity toward butanol increases, selectivity toward biomass decreases, which causes the growth rate of the microbes to become slower. In chemostats, the growth rate limits the dilution rate and, by extension, the productivity of the system. Therefore, while increases in carbon selectivity beyond 0.8 will decrease the material/energy costs of the process, eventually the decreases in productivity will be so substantial that increased capital expenses will overcome any savings in operating costs. Other reactor types, such as submerged membrane bioreactors or biofilm bioreactors, which allow liquids to freely flow through the bioreactor while cells are retained inside,^53,54^ may increase the productivity of EMP systems by decoupling the dilution rate from the cell growth rate. Modeling efforts that examine whether novel bioreactor schemes can improve the performance and economics of EMP processes would be an interesting area of further exploration.

Varying the capital cost of the bioreactor by ±50% can cause changes of ±$1.05 and ±$1.97 to the butanol selling price in the knallgas- and acetogen-based systems, respectively (Figure 5C). The exact cost of the bioreactor in an EMP system is subject to considerable uncertainty. As large-scale EMP systems have yet to be demonstrated, we rely on data for large-scale aerobic fermenters in our analysis. While using these data is appropriate in the current conceptual analysis (due to the fact that both EMP reactors and aerobic reactors are designed around maximizing gas–liquid mass transfer), a more thorough analysis of the cost of bioreactors for large-scale EMP systems will be necessary as the field moves closer to industrial adoption.

The cost of electricity, as expected, is the most important material/energy cost that affects the butanol selling price (Figure 5B). A best-case “free-electricity” scenario reduces the cost of butanol to $5.93/gal ($1.56/L) and $10.18/gal ($2.68/L) for the two systems. The most influential financial parameter examined was the discount rate. A 4% variation in the discount rate causes the butanol selling price to change by about $1/gal for the knallgas bacteria-based system and about $2/gal for the acetogen-based system. Financial assumptions, in addition to technical parameters, can have significant impacts on the butanol selling price.

### Future Outlook of EMP

We have developed a multipart framework to analyze the techno-economics of a hypothetical scaled-up DAC–EMP butanol production process, with two possible variations examined: a one-step bioprocess using a knallgas bacterium and a two-step acetate-mediated bioprocess based on an acetogenic and an acetotrophic microbe. We began by developing physics-based models for the constitutive components that make up the process to predict performance metrics, such as energy efficiency, productivity, and titer, based on limited empirical data. Material and energy demands were then calculated for the process at a given scale, and a techno-economic assessment determined the operating costs, capital costs, and a minimum *n*-butanol selling price. This techno-economic assessment is still quite conceptual in nature, given that it evaluated a proposed DAC–EMP system that has not yet been developed but is instead modeled from first principles. This analysis, however, does contextualize various metrics (e.g., productivity, titer, energy efficiency, material demands) to understand how they impact the viability of DAC–EMP. This analysis suggests that the economics of this process may be challenging, although a road to economic viability is possible if conditions both external and internal to this specific system can be realized.

Beyond engineering efficient EMP systems, this analysis revealed cost factors that affect the system’s economic potential. The assessment presented here indicates that the current cost of renewable hydrogen is too high for this process to be economical. However, assuming that projections of lower hydrogen costs ($1/kg) are realized, the system will not be limited by the cost of electrolysis. The decrease in electrolyzer costs in previous years and the rapidly declining cost of solar electricity production give reason to be optimistic about this aspect.^55,56^ Similarly, the cost of CO_2_ produced through DAC at current prices ($500–600/ton)^19^ would be prohibitively expensive for the EMP process described. However, assuming sufficiently low DAC costs (∼$105/ton in our optimistic projection), the cost of carbon will not limit the system.

If these external cost targets can be met, then economic viability becomes contingent on the performance of the bioprocess itself. The selectivity of butanol production vs biomass formation will be a key factor in determining the cost. The cost of H_2_ will vary inversely with selectivity, and therefore a selectivity close to the theoretical maximum (>80%) is needed for a cost-effective process. The volumetric butanol production rate is another key determining factor of the capital cost of the process, and improving this metric beyond the base-case estimates described earlier will likely be critical for a cost-effective EMP process. In addition, the cost of separations was heavily dependent on titer, especially below 15 g L^–1^ of n-butanol; therefore, microbial engineering strategies to improve the solvent tolerance of EMP-relevant microbes should be pursued.

Due to a variety of factors discussed, the knallgas bacteria-based system appears better poised for commercialization in the short term. This should not discourage research into two-step EMP systems, however, as they do maintain several advantages not captured in this analysis, including reduced safety concerns (as H_2_/O_2_ gas mixtures can be avoided) and the significantly more developed genetic toolkit for acetotrophic microbes such as *E. coli* compared to knallgas bacteria such as *C. necator*.

In summary, this analysis predicts that butanol produced by a knallgas bacteria-based EMP system can only be cost-competitive (<$6/gal) under a set of optimistic assumptions including the costs of hydrogen, DAC, and renewable electricity falling to $1/kg, $105/ton, and $0.02/kW h, respectively; carbon selectivity >80%; productivity >1.5 g BuOH L^–1^ h^–1^; and titer >15 g L^–1^. However, all of these barriers can be addressed. Researchers working on these systems should therefore continue their efforts and focus on creating innovative strategies to address these problems. Finally, the approach described here, combining first-principles modeling and techno-economic assessment, provides a straightforward method to analyze other EMP strategies and applications. While biofuels may face challenges in competing with fossil fuels, other products of higher value may be economically produced through EMP more readily. Other products can easily be examined by varying the stoichiometry and yields described in the model equations. This work details the development of a useful tool for understanding the economics of large-scale commodity chemical production through EMP that can in turn guide future research directions.

## Supporting Information Available

Model equations, tables of parameters and base-case assumptions, assumptions for economic analysis, sensitivity analysis results, process flow diagrams, and supplemental notes (PDF)
