# Supplementary material for: Techno-Economic Assessment of Electromicrobial Production of n-Butanol from Air-Captured CO2
Source: Environ Sci Technol. 2024 Apr 15;58(17):7302–13. doi: 10.1021/acs.est.3c08748 (PMC11064224; doi:10.1021/acs.est.3c08748)
Supplement: Supplementary file 1 — es3c08748_si_001.pdf [file es3c08748_si_001.pdf]

Supporting Information

Techno-economic assessment of electromicrobial production of n-butanol from air-captured CO_2

Authors

Jeremy David Adams,[1] Douglas S. Clark[1,2*]

Affiliations

1. Department of Chemical and Biomolecular Engineering, University of California, Berkeley, Berkeley, CA 94720, USA
2. Molecular Biophysics and Integrated Bioimaging Division, Lawrence Berkeley National Laboratory, 1 Cyclotron Road, Berkeley, CA 94720, USA

[*]Correspondence should be addressed to D.S.C (dsc@berkeley.edu)

Summary: 33 pages, 11 tables, 7 figures

Supporting Information

Note S1: Direct air capture model equations

The DAC contactor is modeled as a monolithic catalyst support with cylindrical channels coated with the sorbent onto which CO_2 reversibly chemisorbs. The concentration of adsorbed carbon dioxide, Q, in units of moles of CO_2 per kg of sorbent, as a function of time (t) and length (z) along the DAC contactor, can be described by the following equation:

$$\frac{\partial Q(z,t)}{\partial t} = k(Q^{\mathrm{eq}}(z, T_{\mathrm{ads}}, p_{\mathrm{CO2}}) - Q(z,t)) \tag{S-1}$$

where *k* is the rate constant of the chemisorption reaction in the forward direction and Q^{eq} is the equilibrium concentration of adsorbed CO_2 at a given partial pressure and temperature. Q^{eq} is modeled here as a step isotherm where Langmuir behavior is exhibited before and after the step (see Note S4 for validation of the model isotherm against literature data):

$$Q^{\mathrm{eq}} = \begin{cases} \dfrac{Q^{\mathrm{sat1}} K_{\mathrm{eq1}} (\frac{p_{\mathrm{CO2}}}{p^\circ})}{1 + K_{\mathrm{eq1}} (\frac{p_{\mathrm{CO2}}}{p^\circ})} & p_{\mathrm{CO2}} < p_{\mathrm{step}} \\ \dfrac{Q^{\mathrm{sat1}} K_{\mathrm{eq1}} (\frac{p_{\mathrm{step}}}{p^\circ})}{1 + K_{\mathrm{eq1}} (\frac{p_{\mathrm{step}}}{p^\circ})} + \dfrac{Q^{\mathrm{sat2}} K_{\mathrm{eq2}} (\frac{p_{\mathrm{CO2}} - p_{\mathrm{step}}}{p^\circ})}{1 + K_{\mathrm{eq2}} (\frac{p_{\mathrm{CO2}} - p_{\mathrm{step}}}{p^\circ})} & p_{\mathrm{CO2}} \geq p_{\mathrm{step}} \end{cases} \tag{S-2}$$

where Q^{sat1} and Q^{sat2} represent the maximum adsorbed CO_2 concentration (moles CO_2 per kg sorbent) associated with each regime, p_{CO2} is the partial pressure of unadsorbed CO_2 in the contactor, p° is standard pressure (1 bar), p_{step} is the partial pressure of CO_2 at which the step occurs, and K_{eq1} and K_{eq2} are the equilibrium constants of the reversible chemisorption reaction associated with each adsorption regime. K_{eq1}, K_{eq2} and p_{step} are themselves functions of the adsorbent temperature T_{ads} and can be described by a Clausius-Clapeyron relationship as:

$$\begin{aligned} K_{\mathrm{eq1}} &= K_{\mathrm{eq1}}(T_{\mathrm{ref}}) \exp\left[\frac{-\Delta H_{ads}}{R}\left(\frac{1}{T_{\mathrm{ads}}} - \frac{1}{T_{\mathrm{ref}}}\right)\right] \\ K_{\mathrm{eq2}} &= K_{\mathrm{eq2}}(T_{\mathrm{ref}}) \exp\left[\frac{-\Delta H_{ads}}{R}\left(\frac{1}{T_{\mathrm{ads}}} - \frac{1}{T_{\mathrm{ref}}}\right)\right] \\ p_{\mathrm{step}} &= p_{\mathrm{step}}(T_{\mathrm{ref}}) \exp\left[\frac{\Delta H_{ads}}{R}\left(\frac{1}{T_{\mathrm{ads}}} - \frac{1}{T_{\mathrm{ref}}}\right)\right] \end{aligned} \tag{S-3}$$

where ΔH_{ads} is the enthalpy of adsorption and T_{ref} is some reference temperature for which the isotherm parameters are known.

The change in the concentration of unadsorbed carbon dioxide within the contactor space (represented as a partial pressure of CO_2), which can vary in the axial dimension z along the length of the contactor (but is assumed to be constant in the radial dimension), is determined by a mass balance to be:

$$\varepsilon \frac{\partial p_{\mathrm{CO2}}}{\partial t} = \frac{L}{\tau}\frac{\partial p_{\mathrm{CO2}}}{\partial z} - kRT_{\mathrm{air}}\rho_{\mathrm{ads}}(1-\varepsilon)(Q^{\mathrm{eq}} - Q) \tag{S-4}$$

where τ is the space time of the air inside the contactor of length L, R is the gas constant, T_{air} is the temperature of the air inside the contactor, ρ_{ads} is the void-free density of the adsorbent, and ε is the void fraction of the contactor.

During the adsorption phase, the temperature of the adsorbent can change in response to two factors: heat transfer to/from the air blowing into the contactor, and heat generated by the chemisorption process. We assume no spatial temperature gradients form in the contactor. Energy balances yield the following equation for the change in temperature of the adsorbent:

$$c_{\mathrm{p,T}}\frac{dT_{\mathrm{ads}}}{dt}=\frac{2h_{\mathrm{A}}}{r\rho_{\mathrm{ads}}}\frac{T_{\mathrm{air}}-T_{\mathrm{ads}}}{1-\varepsilon}-\Delta H_{\mathrm{ads}}\frac{1}{L}\int_{z=0}^{z=L}\frac{\partial Q(z,t)}{\partial t}dz \tag{S-5}$$

where h_{A} is the overall heat transfer coefficient between air and the adsorbent/monolithic support and r is the radius of the cylindrical channels in the monolith. We also define a total heat capacity $c_{\mathrm{p,T}}$ as the energy required to raise 1 kg of the sorbent 1 °C, given that the monolithic structure and adsorbed CO_2 must also be raised by 1 °C, calculated as:

$$c_{\mathrm{p,T}}=c_{\mathrm{p,ads}}+c_{\mathrm{p,mon}}\frac{m_{\mathrm{mon}}}{m_{\mathrm{ads}}}+\hat{c}_{\mathrm{p,CO2}}Q \tag{S-6}$$

where $c_{\mathrm{p,ads}}$, $c_{\mathrm{p,mon}}$, $\hat{c}_{\mathrm{p,CO2}}$ are specific heat capacities of the adsorbent, monolithic support, and CO_2, respectively (note: $\hat{c}_{\mathrm{p,CO2}}$ is on a molar basis), and $\frac{m_{\mathrm{mon}}}{m_{\mathrm{ads}}}$ describes the ratio of the mass of the monolithic support and the mass of the adsorbent.

Similarly, energy balances on the air inside the contactor can be written, again assuming no spatial temperature gradients for air in the contactor emerge, yielding the expression:

$$\frac{dT_{\mathrm{air}}}{dt}=-\frac{2h_{A}}{r\rho_{\mathrm{air}}}\frac{T_{\mathrm{air}}-T_{\mathrm{ads}}}{c_{\mathrm{p,air}}\varepsilon}+\frac{T_{0}-T_{\mathrm{air}}}{\tau\varepsilon} \tag{S-7}$$

where ρ_{air} is the density of air (calculated by the ideal gas law), $c_{\mathrm{p,air}}$ is the constant-pressure heat capacity of air, and T_0 is the temperature of the incoming ambient air.

The desorption model equations can then be written. During desorption, air flow into the contactor stops, the inlet is sealed, and heat (via steam) is applied to the walls of the contactor channels. Vacuum is applied to the outlet of the contactor to remove CO_2 as it desorbs. When considering the desorption process, the same equations used in the adsorption process hold with minor modifications. First, in Eqn. S-1 and S-4, there are no longer spatial gradients to the concentration of CO_2, either adsorbed or in the gas phase. The change in adsorbed concentration in the reactor is therefore modeled as:

$$\frac{dQ(t)}{dt}=k(Q^{\mathrm{eq}}(T_{\mathrm{ads}},p_{\mathrm{CO2}})-Q(t)) \tag{S-8}$$

Moreover, in this part of the process, there is no flow of gas into the contactor. However, there is flow of the CO_2 stream out of the contactor due to the applied vacuum. The equation for describing the change of partial pressure of CO_2 inside the contactor is therefore rewritten as:

$$\varepsilon\frac{dp_{\mathrm{CO2}}}{dt}=-\frac{p_{\mathrm{CO2}}}{L}\sqrt{\frac{2\gamma}{\gamma-1}\frac{p_{\mathrm{CO2}}}{\rho_{\mathrm{CO2}}}(1-\frac{p_{\mathrm{vac}}}{p_{\mathrm{CO2}}})^{\frac{\gamma-1}{\gamma}}}-kRT_{\mathrm{CO2}}\rho_{\mathrm{ads}}(1-\varepsilon)(Q^{\mathrm{eq}}-Q) \tag{S-9}$$

where p_{vac} is the applied vacuum pressure, γ is the ratio of constant-pressure and constant-volume heat capacities of CO_2, and ρ_{CO2} is the density of CO_2, as approximated by the ideal gas law.

The energy balance on the adsorbent is also adjusted to remove the spatial gradient and is rewritten as:

$$c_{\mathrm{p,T}}\frac{dT_{\mathrm{ads}}}{dt}=\frac{2h_{s}}{r\rho_{\mathrm{ads}}}\frac{T_{\mathrm{s}}-T_{\mathrm{ads}}}{1-\varepsilon}-\Delta H_{\mathrm{ads}}\frac{dQ}{dt} \tag{S-10}$$

where h_s is the overall heat transfer coefficient for transferring heat from steam to the contactor and T_{s} is the temperature of steam. It is assumed that the CO_2 will leave the reactor at the temperature of the heating steam.

Note S2: Bioprocess model equations

Two hypothetical EMP systems are considered to convert H_2 and CO_2 to n-butanol. The Knallgas bacteria-based system consists of a bioreactor containing a Knallgas bacterium (*e.g.*, *Cupriavidus necator*) which converts CO_2, H_2, and O_2 (each of which is bubbled into the bioreactor) into biomass and n-butanol. The other system considered, the acetogen-based system, consists of two bioreactors. CO_2 and H_2 are continuously bubbled into the first bioreactor and are converted to biomass and acetic acid by an acetogen (*e.g.*, *Sporomusa ovata*) under anaerobic conditions. This acetate-rich medium is fed to a second bioreactor containing an acetotrophic microbe (*e.g.*, *E. coli*) that converts the acetate to biomass and n-butanol under aerobic conditions. All bioreactors in both systems are continuously fed minimal medium, containing ammonium and other necessary nutrients, allowing for continuous operation (*i.e.*, chemostat).

Throughout the model equations, the subscript K will be used to denote the variables/parameters in the bioreactor of the Knallgas bacteria-based system, while the subscripts $\mathrm{A},1$ and $\mathrm{A},2$ will be used to denote the variables/parameters in the first and second bioreactor of the acetogen-based system, respectively.

<u>Knallgas bacteria-based system model equations:</u>

It is assumed that all carbon fixed by the Knallgas bacteria either becomes n-butanol (Bu) or biomass (X). The chemical equation for the production of biomass in this system is:

$$\kappa_{\mathrm{X,H_2,K}}\mathrm{H_2} + \kappa_{\mathrm{X,CO_2,K}}\mathrm{CO_2} + \kappa_{\mathrm{X,O_2,K}}\mathrm{O_2} + \kappa_{\mathrm{X,NH_3,K}}\mathrm{NH_3} \rightarrow \mathrm{CH_{1.77}O_{0.49}N_{0.24}} + \kappa_{\mathrm{X,H_2O,K}}\mathrm{H_2O} \quad \text{(S-11)}$$

where $\kappa_{i,j,\mathrm{K}}$ is the stoichiometric coefficient of species j in the equation for the formation of product i. An empirical molar yield of biomass on hydrogen is used to determine the value of $\kappa_{\mathrm{X,H_2,K}}$, while the rest of the coefficients are obtained by balancing the chemical equation. The equation for the production of n-butanol is similarly written:

$$\kappa_{\mathrm{Bu,H_2,K}}\mathrm{H_2} + \kappa_{\mathrm{Bu,CO_2,K}}\mathrm{CO_2} + \kappa_{\mathrm{Bu,O_2,K}}\mathrm{O_2} \rightarrow \mathrm{C_4H_9OH} + \kappa_{\mathrm{Bu,H_2O,K}}\mathrm{H_2O} \quad \text{(S-12)}$$

The stoichiometric coefficients for this equation are determined by considering the metabolic pathway by which a Knallgas bacterium would produce n-butanol. Carbon dioxide would be fixed by the Calvin cycle:

$$3\mathrm{CO_2} + 5\mathrm{NADH} + 7(\mathrm{ATP} + \mathrm{H_2O}) + 4\mathrm{H^+} \rightarrow \mathrm{C_3H_3O_3^-} + 5\mathrm{NAD^+} + 7(\mathrm{ADP} + \mathrm{P_i}) + 3\mathrm{H_2O} \quad \text{(S-13)}$$

The necessary NADH for the Calvin cycle and further redox reactions can be generated from hydrogenases:

$$\mathrm{H_2} + \mathrm{NAD^+} \rightarrow \mathrm{H^+} + \mathrm{NADH} \quad \text{(S-14)}$$

and oxidative phosphorylation can be used to aerobically generate ATP from the produced NADH:

$$\mathrm{NADH} + \mathrm{H^+} + \frac{1}{2}\mathrm{O_2} + \left(\frac{\mathrm{P}}{\mathrm{O}}\right)(\mathrm{ADP} + \mathrm{P_i}) \rightarrow \mathrm{NAD^+} + \mathrm{H_2O} + \left(\frac{\mathrm{P}}{\mathrm{O}}\right)(\mathrm{ATP} + \mathrm{H_2O}) \quad \text{(S-15)}$$

Pyruvate produced from the Calvin cycle can then be decarboxylated to produce acetyl-CoA:

$$\mathrm{C_3H_3O_3^-} + \mathrm{NAD^+} + \mathrm{HSCoA} \rightarrow \mathrm{C_2H_3OSCoA} + \mathrm{NADH} + \mathrm{CO_2} \quad \text{(S-16)}$$

Two acetyl-CoA molecules can react to form acetoacetyl-CoA, which through a series of enzymatic steps can be reduced to n-butanol:

$$2\mathrm{C_2H_3OSCoA} + 4\mathrm{NADH} + 4\mathrm{H^+} \rightarrow \mathrm{C_4H_9OH} + 4\ \mathrm{NAD^+} + \mathrm{H_2O} + 2\mathrm{HSCoA} \quad \text{(S-17)}$$

Assuming a P/O ratio of 2.5, the above equations can be linearly combined to yield:

$$4\mathrm{CO_2} + 17.6\mathrm{H_2} + 2.8\mathrm{O_2} \rightarrow \mathrm{C_4H_9OH} + 12.6\mathrm{H_2O} \quad \text{(S-18)}$$

The specific growth rate of the Knallgas bacterium (μ_K) is a function of the concentration of hydrogen ($c_{\mathrm{H_2,K}}$), carbon dioxide ($c_{\mathrm{CO_2,K}}$), and oxygen ($c_{\mathrm{O_2,K}}$) in the bioreactor:

$$\mu_{\mathrm{K}}=\frac{\mu_{\mathrm{K,max}}c_{\mathrm{H_2,K}}c_{\mathrm{CO_2,K}}c_{\mathrm{O_2,K}}}{(K_{\mathrm{H_2,K}}+c_{\mathrm{H_2,K}})(K_{\mathrm{CO_2,K}}+c_{\mathrm{CO_2,K}})(K_{\mathrm{O_2,K}}+c_{\mathrm{O_2,K}})} \tag{S-19}$$

where $K_{\mathrm{H_2,K}}$, $K_{\mathrm{CO_2,K}}$, and $K_{\mathrm{O_2,K}}$ are Monod constants for H_2, CO_2, and O_2, respectively, for the Knallgas bacteria and $\mu_{\mathrm{K,max}}$ is the maximum specific growth rate under optimal conditions. We assume that this growth rate expression represents the total production rate of biomass and n-butanol on a carbon-mole basis. We introduce a selectivity variable ϕ_{K}, which represents the fraction of fixed carbon present in biomass compared to the total carbon present in biomass and n-butanol. The concentration of biomass in the Knallgas bacteria bioreactor (X_{K}) as a function of time is given by:

$$\frac{dX_{\mathrm{K}}}{dt}=X_{\mathrm{K}}(\mu_{\mathrm{K}}\phi_{\mathrm{K}}-D_{\mathrm{K}}) \tag{S-20}$$

where D_{K} is the liquid-phase dilution rate in the Knallgas system. Note that X_{K} is in units of moles of carbon embodied in biomass per liter.

The molar concentration of n-butanol in the reactor $c_{\mathrm{Bu,K}}$ is similarly written as:

$$\frac{dc_{\mathrm{Bu,K}}}{dt}=\mu_{\mathrm{K}}\frac{1-\phi_{\mathrm{K}}}{4}X_K-D_Kc_{\mathrm{Bu,K}} \tag{S-21}$$

where the 4 accounts for the fact that there are 4 moles of carbon atoms per mole of butanol. We define a maximum butanol concentration parameter ($c_{\mathrm{Bu,max,K}}$) to account for effects of butanol toxicity. If the butanol concentration exceeds this maximum value, the growth rate of the bacteria is set to zero.

The concentration of each of the dissolved gas species in the liquid phase of the bioreactor can be modeled. For example, hydrogen enters the media through gas-liquid mass transfer, is consumed by the production of biomass or n-butanol, and exits the bioreactor in the liquid effluent. Mass balances then yield the following expression for the concentration of dissolved hydrogen in the Knallgas bioreactor ($c_{\mathrm{H_2,K}}$):

$$\frac{dc_{\mathrm{H_2,K}}}{dt}=k_{\mathrm{L}}a_{\mathrm{H_2,K}}\left(H_{\mathrm{H_2}}p_{\mathrm{H_2,K}}-c_{\mathrm{H_2,K}}\right)-\mu_{\mathrm{K}}X_{\mathrm{K}}\left(\phi_{\mathrm{K}}\kappa_{\mathrm{X,H_2,K}}+\frac{(1-\phi_{\mathrm{K}})}{4}\kappa_{\mathrm{Bu,H_2,K}}\right)-D_Kc_{\mathrm{H_2,K}} \tag{S-22}$$

where $k_{\mathrm{L}}a_{\mathrm{H_2,K}}$ is the volumetric gas-liquid mass transfer coefficient for H_2 into the medium, $H_{\mathrm{H_2}}$ is the Henry's Law constant for hydrogen gas in water, and $p_{\mathrm{H_2,K}}$ is the partial pressure of hydrogen in the gas phase of the bioreactor. By analogy, the concentration of dissolved oxygen ($c_{\mathrm{O_2,K}}$) is written as:

$$\frac{dc_{O_2,K}}{dt}=k_La_{O_2,K}\left(H_{O_2}p_{O_2,K}-c_{O_2,K}\right)-\mu_KX_K\left(\phi_K\kappa_{X,O_2,K}+\frac{(1-\phi_K)}{4}\kappa_{Bu,O_2,K}\right)-D_Kc_{O_2,K} \tag{S-23}$$

and the concentration of dissolved carbon dioxide is written as:

$$\frac{dc_{\mathrm{CO_2,K}}}{dt}=k_{\mathrm{L}}a_{\mathrm{CO_2,K}}\left(H_{\mathrm{O_2}}p_{\mathrm{CO_2,K}}-c_{\mathrm{CO_2,K}}\right)-\mu_{\mathrm{K}}X_{\mathrm{K}}\left(\phi_{\mathrm{K}}\kappa_{\mathrm{X,CO_2,K}}+\frac{(1-\phi_{\mathrm{K}})}{4}\kappa_{\mathrm{Bu,CO_2,K}}\right)-D_Kc_{\mathrm{CO_2,K}} \tag{S-24}$$

In addition to the liquid flow in the bioreactor, the gaseous substrates are continuously bubbled through the bioreactor, and a constant volumetric flow rate of gases in and out of the bioreactor is assumed. The partial pressure of each of the gaseous substrates in the headspace is modeled. For example, the partial pressure of hydrogen in the headspace of the Knallgas bioreactor ($p_{\mathrm{H_2,K}}$) is:

$$\frac{dp_{\mathrm{H_2,K}}}{dt}=D_{\mathrm{gas,K}}(p_{\mathrm{H_2},i,\mathrm{K}}-p_{\mathrm{H_2,K}})-\frac{V_{\mathrm{L,K}}}{V_{\mathrm{G,K}}}RT_{\mathrm{K}}k_{\mathrm{L}}a_{\mathrm{H_2,K}}\left(H_{\mathrm{H_2}}p_{\mathrm{H_2,K}}-c_{\mathrm{H_2,K}}\right) \tag{S-25}$$

where $D_{\mathrm{gas,K}}$ is the gas-phase dilution rate (total gas volumetric flow rate divided by headspace volume), $p_{\mathrm{H_2},i,\mathrm{K}}$ is the partial pressure of hydrogen in the gas feed, $\frac{V_{\mathrm{L,K}}}{V_{\mathrm{G,K}}}$ is the ratio of the working liquid volume to the headspace volume in the bioreactor, R is the gas constant, and T_{K} is the temperature of the Knallgas bioreactor. By analogy, the partial pressure of oxygen in the headspace ($p_{\mathrm{O_2,K}}$) is:

$$\frac{dp_{O_2,K}}{dt} = D_{gas,K}(p_{O_2,i,K} - p_{O_2,K}) - \frac{V_{L,K}}{V_{G,K}} RT_K k_L a_{O_2,K}\left(H_{O_2} p_{O_2,K} - c_{O_2,K}\right) \quad \text{(S-26)}$$

and the partial pressure of carbon dioxide ($p_{CO_2,K}$) is:

$$\frac{dp_{CO_2,K}}{dt} = D_{gas,K}(p_{CO_2,i,K} - p_{CO_2,K}) - \frac{V_{L,K}}{V_{G,K}} RT_K k_L a_{CO_2,K}\left(H_{CO_2} p_{CO_2,K} - c_{CO_2,K}\right) \quad \text{(S-27)}$$

Acetogen-based system model equations:

The acetogen fixes CO_2 through the Wood-Ljungdahl Pathway, using H_2 as an energy source, and produces acetic acid:

$$4H_2 + 2CO_2 \rightarrow CH_3COOH + 2H_2O \quad \text{(S-28)}$$

This acetate generation provides energy used to produce biomass (the same chemical formula for biomass, $CH_{1.77}O_{0.49}N_{0.24}$, is used as before). The ratio of acetate to biomass formation is dependent on the level of energy conservation, represented by an ATP conservation coefficient, or the number of ATP molecules generated by proton/sodium gradients for each acetyl-CoA molecule formed through the Wood-Ljungdahl pathway.

We have previously described the overall stoichiometry of biomass growth and acetate production of acetogenic microbes,[1] following the methodology of Fast and Papoutsakis,[2] assuming a reasonable ATP conservation coefficient of 0.47:[3]

$$38.33H_2 + 19.15CO_2 + 0.24NH_3 \rightarrow CH_{1.77}O_{0.49}N_{0.24} + 9.07CH_3COOH + 19.66H_2O \quad \text{(S-29)}$$

The acetogenic bioreactor (denoted as $A, 1$) is modeled as a chemostat, with a dilution rate of $D_{A,1}$. Assuming a well-mixed reactor environment of constant volume, the concentration of cells in the bioreactor, $X_{A,1}$, as a function of time is written as:

$$\frac{dX_{A,1}}{dt} = (\mu_{A,1} - D_{A,1})X_{A,1} \quad \text{(S-30)}$$

As was the case in the Knallgas bacteria-based system, the biomass concentration is expressed in terms of the molar concentration of carbon that is embodied in biomass in the reactor. Unlike the previous case, however, the specific growth rate only represents the biomass formation rate. Assuming that hydrogen and carbon dioxide are the major substrates for the acetogen, Monod kinetics are used to predict the growth rate, $\mu_{A,1}$:

$$\mu_{A,1} = \frac{\mu_{max,A,1}\sigma_{A,1}\left(c_{Na,A,1}\right)c_{H_2,A,1}c_{CO_2,A,1}}{(K_{H_2,A,1} + c_{H_2,A,1})(K_{CO_2,A,1} + c_{CO_2,A,1})} \quad \text{(S-31)}$$

where $c_{H_2,A,1}$ is the molar concentration of dissolved hydrogen, $c_{CO_2,A,1}$ is the molar concentration of dissolved CO_2, $c_{Na,A,1}$ is the molar concentration of sodium, $K_{H_2,A,1}$ is the Monod constant for hydrogen associated with acetogenic growth, $K_{CO_2,A,1}$ is the Monod constant for CO_2 for the acetogen, and $\mu_{max,A,1}$ is the maximum specific growth rate of the acetogen under optimal conditions. The model also takes into account the effect of salinity, introducing a factor $\sigma_{A,1}$ that adjusts the growth rate and depends on the sodium concentration in the bioreactor medium ($c_{Na,A,1}$):

$$\sigma_{A,1} = \begin{cases} 1 & c_{Na,A,1} < c_{Na,min,A,1} \\ 1 - \dfrac{c_{Na,A,1}}{c_{Na,max,A,1} - c_{Na,min,A,1}} & c_{Na,min,A,1} < c_{Na,A,1} < c_{Na,max,A,1} \\ 0 & c_{Na,A,1} > c_{Na,max,A,1} \end{cases} \quad \text{(S-32)}$$

Because NaOH is added to neutralize the acetic acid generated by the acetogen, the molar concentration of sodium in the acetogen-containing bioreactor is equal to the molar concentration of acetate.

Based on the stoichiometry described in Eqn. S-29, the concentration of acetate in the acetogenic bioreactor ($c_{\mathrm{Ac,A,1}}$) is described as:

$$\frac{dc_{\mathrm{Ac,A,1}}}{dt} = 9.07\mu_{\mathrm{A,1}}X_{\mathrm{A,1}} - D_{\mathrm{A,1}}c_{\mathrm{Ac,A,1}} \tag{S-33}$$

Dissolved hydrogen ($c_{\mathrm{H2,1}}$) is transferred into the liquid phase from the gas phase and is consumed by the acetogen, again following the stoichiometry described in Eqn. S-29:

$$\frac{dc_{\mathrm{H_2,A,1}}}{dt} = k_{\mathrm{L}}a_{\mathrm{H_2,A,1}}\left(H_{\mathrm{H_2}}p_{\mathrm{H_2,A,1}} - c_{\mathrm{H_2,A,1}}\right) - 38.33\mu_{\mathrm{A,1}}X_{\mathrm{A,1}} - D_{\mathrm{A,1}}c_{\mathrm{H_2,A,1}} \tag{S-34}$$

while the partial pressure of hydrogen in the head space $p_{\mathrm{H_2,A,1}}$ is:

$$\frac{dp_{\mathrm{H_2,A,1}}}{dt} = \left(p_{\mathrm{H_2},i,\mathrm{A,1}} - p_{\mathrm{H_2,A,1}}\right)D_{\mathrm{gas,A,1}} - RT_{\mathrm{A,1}}k_{\mathrm{L}}a_{\mathrm{H_2,A,1}}\left(H_{\mathrm{H_2}}p_{\mathrm{H_2,A,1}} - c_{\mathrm{H_2,A,1}}\right)\frac{V_{\mathrm{L,A,1}}}{V_{\mathrm{G,A,1}}} \tag{S-35}$$

where all variables and parameters follow the same notation as in Eqn. S-22 and S-25.

By analogy, the dissolved concentration of CO_2 ($c_{\mathrm{CO_2,A,1}}$) is:

$$\frac{dc_{\mathrm{CO_2,A,1}}}{dt} = k_{\mathrm{L}}a_{\mathrm{CO_2,A,1}}\left(H_{\mathrm{CO_2}}p_{\mathrm{CO_2,A,1}} - c_{\mathrm{CO_2,A,1}}\right) - 19.15\mu_{\mathrm{A,1}}X_{\mathrm{A,1}} - D_{\mathrm{A,1}}c_{\mathrm{CO_2,A,1}} \tag{S-36}$$

and the partial pressure of CO_2 in the headspace ($p_{\mathrm{CO_2,A,1}}$) is:

$$\frac{dp_{\mathrm{CO_2,A,1}}}{dt} = \left(p_{\mathrm{CO_2},i,\mathrm{A,1}} - p_{\mathrm{CO_2,A,1}}\right)D_{\mathrm{gas,A,1}} - RT_{\mathrm{A,1}}k_{\mathrm{L}}a_{\mathrm{CO_2,A,1}}\left(H_{\mathrm{CO_2}}p_{\mathrm{CO_2,A,1}} - c_{\mathrm{CO_2,A,1}}\right)\frac{V_{\mathrm{L,A,1}}}{V_{\mathrm{G,A,1}}} \tag{S-37}$$

where all variables and parameters have the same meaning, except for CO_2 instead of H_2.

The second bioreactor in the acetogen-based system $(\mathrm{A},2)$, which converts acetate to n-butanol, is similarly modeled as a chemostat. The acetotroph in the second bioreactor can convert acetate into biomass or into n-butanol. The chemical equation for conversion of acetate into biomass is:

$$\begin{aligned}\kappa_{\mathrm{X,Ac,A,2}}\mathrm{CH_3COOH} + \kappa_{\mathrm{X,O_2,A,2}}\mathrm{O_2} + \kappa_{\mathrm{X,NH_3,A,2}}\mathrm{NH_3} \\ \rightarrow \mathrm{CH_{1.77}O_{0.49}N_{0.24}} + \kappa_{\mathrm{X,CO_2,A,2}}\mathrm{CO_2} + \kappa_{\mathrm{X,H_2O,A,2}}\mathrm{H_2O}\end{aligned} \tag{S-38}$$

where $\kappa_{i,j,\mathrm{A,2}}$ is the stoichiometric coefficient for the species j in the equation for the formation of species i for the acetotrophic bacteria. As with the Knallgas bacteria biomass formation equation, $\kappa_{\mathrm{X,Ac,A,2}}$ can be determined by an empirical molar yield of biomass on acetate, with other coefficients determined by balancing the chemical equation.

The chemical formula for the conversion of acetate into n-butanol is:

$$\kappa_{\mathrm{Bu,Ac,A,2}}\mathrm{CH_3COOH} + \kappa_{\mathrm{Bu,O_2,A,2}}\mathrm{O_2} \rightarrow \mathrm{C_4H_9OH} + \kappa_{\mathrm{Bu,CO_2,A,2}}\mathrm{CO_2} + \kappa_{\mathrm{Bu,H_2O,A,2}}\mathrm{H_2O} \tag{S-39}$$

The stoichiometric coefficients for this equation are determined by considering the metabolic pathway that can produce n-butanol from acetate. Acetotrophs first upgrade acetate to acetyl-CoA using ATP as an energy source.

$$\mathrm{CH_3COOH} + 2(\mathrm{ATP} + \mathrm{H_2O}) + \mathrm{HSCoA} \rightarrow \mathrm{C_2H_3OSCoA} + 2(\mathrm{ADP} + \mathrm{P_i}) + \mathrm{H_2O} \tag{S-40}$$

Note: acetyl-CoA synthetase utilizes ATP as a substrate and produces AMP to drive the ligation of CoA and acetate; due to the fast recombination of AMP and ATP to form two ADP molecules, the chemical equation is simplified by including two ATP→ADP reactions. As before, n-butanol can be produced from acetyl-CoA and NADH through a series of enzymatic reactions:

$$2\mathrm{C_2H_3OSCoA} + 4\mathrm{NADH} + 4\mathrm{H^+} \rightarrow \mathrm{C_4H_9OH} + 4\,\mathrm{NAD^+} + \mathrm{H_2O} + 2\mathrm{HSCoA} \tag{S-41}$$

To provide the necessary ATP and NADH molecules required for the previous two steps, some of the acetyl-CoA will be consumed in the TCA cycle:

$$\begin{aligned} &C_2H_3OSCoA + 3NAD^+ + FAD + ADP + P_i + 2H_2O \\ &\qquad \rightarrow HSCoA + 3NADH + FADH_2 + 3H^+ + ATP + 2CO_2 \end{aligned} \quad \text{(S-42)}$$

Additional ATP can be generated by the oxidative phosphorylation using NADH:

$$NADH + H^+ + \frac{1}{2}O_2 + \left(\frac{P}{O}\right)(ADP + P_i) \rightarrow NAD^+ + H_2O + \left(\frac{P}{O}\right)(ATP + H_2O) \quad \text{(S-43)}$$

or $FADH_2$:

$$FADH_2 + H^+ + \frac{1}{2}O_2 + \left(\frac{P}{O}\right)' (ADP + P_i) \rightarrow FAD + H_2O + \left(\frac{P}{O}\right)(ATP + H_2O) \quad \text{(S-44)}$$

Assuming an oxidative phosphorylation ratio of 2.5 for NADH $\left(\frac{P}{O}\right)$ and 1.5 for $FADH_2$ $\left(\frac{P}{O}\right)'$, the above equations can be linearly combined to yield:

$$3.75CH_3COOH + 1.5O_2 \rightarrow C_4H_9OH + 3.5CO_2 + 2.5H_2O \quad \text{(S-45)}$$

Like the Knallgas bacteria bioreactor, the selectivity of biomass production over n-butanol production is a variable in modeling the second bioreactor of the acetogen-based system. The variable $\phi_{A,2}$ is introduced as the moles of carbon in biomass divided by the total moles of carbon in both biomass and n-butanol. Therefore, the cell production rate is modified by this ratio, such that the concentration of biomass in the acetotrophic bioreactor ($X_{A,2}$) as a function of time is described as:

$$\frac{dX_{A,2}}{dt} = (\mu_{A,2}\phi_{A,2} - D_{A,2})X_{A,2} \quad \text{(S-46)}$$

where $D_{A,2}$ is the liquid-phase dilution rate of this bioreactor. The growth rate of the acetotrophic microbe ($\mu_{A,2}$), which consumes acetate and oxygen as primary substrates, is:

$$\mu_{A,2} = \frac{\mu_{max,A,2}\sigma_{A,2}\left(c_{Na,A,2}\right)c_{Ac,A,2}c_{O_2,A,2}}{(K_{Ac,A,2} + c_{Ac,A,2} + \frac{c_{Ac,A,2}{}^2}{K_I})(K_{O_2,A,2} + c_{O_2,A,2})} \quad \text{(S-47)}$$

where $\mu_{max,A,2}$ is the maximum growth rate of the acetotroph, $c_{Ac,A,2}$ and $c_{O2,A,2}$ are the concentrations of acetate and oxygen respectively, $K_{Ac,A,2}$ and $K_{O_2,A,2}$ are the Monod constants for acetate and oxygen, and K_I is a substrate inhibition constant for acetate.

The term $\sigma_{A,2}\left(c_{Na,A,2}\right)$ is an analogous function of the form described in Eqn. S-32 (though with potentially different values of $c_{Na,min}$ and $c_{Na,max}$). In the second bioreactor, pH control, to offset the rise in pH when acetate is consumed, is mediated by sulfuric acid. Therefore, this pH control does not increase the concentration of sodium, which is constant in the second bioreactor.

The concentration of n-butanol in the bioreactor ($c_{Bu,A,2}$) can then be described by the expression:

$$\frac{dc_{Bu,A,2}}{dt} = \mu_{A,2}\frac{1 - \phi_{A,2}}{4}X_{A,2} - D_{A,2}c_{Bu,A,2} \quad \text{(S-48)}$$

We define a maximum butanol concentration parameter ($c_{Bu,max,A,2}$) to account for effects of butanol toxicity, as before.

The concentration of acetate in the second bioreactor, which is fed from the effluent of the first bioreactor is:

$$\frac{dc_{Ac,A,2}}{dt} = D_{A,2}\left(c_{Ac,A,1} - c_{Ac,A,2}\right) - \mu_{A,2}X_{A,2}\left(\phi_{A,2}\kappa_{X,Ac,A,2} + \frac{1 - \phi_{A,2}}{4}\kappa_{Bu,Ac,A,2}\right) \quad \text{(S-49)}$$

The partial pressure of oxygen in the bioreactor headspace ($p_{O_2,A,2}$) is described as:

$$\frac{dp_{O_2,A,2}}{dt} = (p_{O_2,i,A,2} - p_{O_2,A,2})D_{gas,A,2} - RT_{A,2}k_L a_{O_2,A,2}(H_{O_2}p_{O_2,A,2} - c_{O_2,A,2})\frac{V_{L,A,2}}{V_{G,A,2}} \quad \text{(S-50)}$$

where the nomenclature of previous equations is maintained.

The concentration of dissolved oxygen in the bioreactor is described by the expression:

$$\frac{dc_{O_2,A,2}}{dt} = k_L a_{O_2,A,2}(H_{O_2}p_{O_2,A,2} - c_{O_2,A,2}) - \mu_{A,2}X_{A,2}\left(\phi_{A,2}\kappa_{X,O_2,A,2} + \frac{1-\phi_{A,2}}{4}\kappa_{Bu,O_2,A,2}\right) - D_{A,2}c_{O_2,A,2} \quad \text{(S-51)}$$

Note S3: Process modeling energy demand equations

The electricity required for the industrial fans (E_1) per mole of CO_2 captured can be calculated as:

$$E_1 = \frac{t_{ads}\Delta P}{\eta_1 \tau\, \Delta q_{cycle}\rho_{ads}(1-\varepsilon)} \quad \text{(S-52)}$$

where η_1 is the efficiency of the fan, t_{ads} is the adsorption time per cycle, and Δq_{cycle} is the moles of CO_2 captured per mass of adsorbent each cycle. ΔP is the pressure drop across the contactor, which is a function of the air velocity and is estimated by the Hagen-Poiseuille equation:

$$\Delta P = \frac{8\mu_{air}L^2}{r^2\tau} \quad \text{(S-53)}$$

where μ_{air} is the dynamic viscosity of air.

During the desorption cycle, the air inside the contactor is first displaced by nitrogen gas to remove oxygen from the product stream. The contactor is maintained at a pressure of P_{vac} by a vacuum pump, which then pressurizes the captured CO_2 to P_2. This is modeled as an adiabatic compression from P_{vac} to P_2, adjusted by a pump efficiency (η_2), and the energy required per mole of CO_2 captured is:

$$E_2 = \frac{1}{\eta_2 y_{CO2}}\frac{RT_0}{\gamma - 1}\left[\left(\frac{P_2}{P_{vac}}\right)^{(\gamma-1)/\gamma} - 1\right] \quad \text{(S-54)}$$

where γ is the ratio of constant-pressure and constant-volume heat capacities of CO_2. The purity of this CO_2 stream, y_{CO2}, is determined by the relative amounts of CO_2 captured and N_2 in the void space:

$$y_{CO2} = \frac{\Delta q_{cycle}\rho_{ads}(1-\varepsilon)RT_0}{\Delta q_{cycle}\rho_{ads}(1-\varepsilon)RT_0 + \varepsilon P_{vac}} \quad \text{(S-55)}$$

Assuming the energy required for the desorption step is provided by steam generated by a heat pump with a defined coefficient of performance (COP), the electricity required for the temperature swing, per mole of CO_2 captured, is calculated as:

$$E_3 = \frac{1}{\eta_3 \mathrm{COP}}\left(\frac{c_{p,T}(T_s - T_0)}{\Delta q_{cycle}} - \Delta H_{ads}\right) \quad \text{(S-56)}$$

where η_3 is an efficiency to account for heat loss to the surroundings.

The electricity required for electrolysis per mole of H_2 is simply calculated as:

$$E_4 = \frac{\Delta H_{comb,H2}}{\eta_4} \quad \text{(S-57)}$$

where $\Delta H_{comb,H2}$ is the enthalpy of combustion of hydrogen and η_4 is the efficiency of the electrolyzer.

The energy demand for bioreactor operation includes the power required to achieve a desired gas-liquid mass transfer coefficient (P_{G}) and the energy required to heat the media from ambient temperature to the desired operating temperature (P_{th}).

The power to achieve a certain $k_{\mathrm{L}}a$ (for oxygen) can be determined from the correlation developed by Vasconcelos *et al.* for stirred tank reactors with a height that is twice the diameter:[4]

$$k_{\mathrm{L}}a_{\mathrm{O_2}} = 22.3(P_{\mathrm{G}})^{0.66}(u_{\mathrm{G}})^{0.51} \tag{S-58}$$

where P_{G} is the specific power input (in units W m^{-3}) and u_{G} is the superficial gas velocity (in units m s^{-1}), which is related to the gas phase dilution rate using:

$$u_{\mathrm{G}} = \frac{D_{\mathrm{gas}}}{A_{\mathrm{S}}} \tag{S-59}$$

where A_{S} is the ratio of the surface area of the sparging holes to the reactor volume. The relationship between the gas-liquid mass transfer coefficient for different gas species under identical sparging/agitation conditions, following the method of Meraz *et al.*,[5] is:

$$k_{\mathrm{L}}a_{i\neq \mathrm{O_2}} = \sqrt{\frac{D_i}{D_{\mathrm{O_2}}}}\, k_{\mathrm{L}}a_{\mathrm{O_2}} \tag{S-60}$$

where D_i is the diffusivity of species i.

The power to heat the bioreactor to the desired temperature is:

$$P_{\mathrm{th},n} = \frac{D_n c_{\mathrm{P,W}} \rho_{\mathrm{W}} (T_n - T_0)}{\mathrm{COP}} \tag{S-61}$$

where D_n is the liquid-phase dilution rate for a given bioreactor, $c_{\mathrm{P,W}}$ is the heat capacity of water, ρ_{W} is the density of water, T_n is the operating temperature of a given bioreactor, and T_0 is the ambient temperature. It is assumed that a heat pump is used to generate the thermal energy for the bioreactor operation, and therefore the specific power term includes a coefficient of performance to calculate the electrical energy requirement.

The bioreactor energy demand per kg of n-butanol produced (E_5) is therefore:

$$E_5 = \frac{P_{\mathrm{th,n}} + \sum P_{\mathrm{G,n}}}{D_n c_{\mathrm{Bu},n} M_{\mathrm{Bu}}} \tag{S-62}$$

where M_{Bu} is the molar mass of n-butanol. The summation term denotes that, for the acetogenic system, the total bioreactor energy demand will include the sum of the power demands for both bioreactors.

Note S4: Validation of model isotherm against literature data

Many of the DAC model parameters are based on the MOF mmen-Mg_2(dobpdc), originally synthesized by McDonald *et al.*[6] The isotherms of this MOF exhibit step-behavior, and the authors fit experimental data to a multi-site Langmuir-Freuidlich model, which was valid across a wide range of temperatures and pressures.

To simplify the DAC model, the adsorption thermodynamics are described by two successive Langmuir isotherms with a transition at a certain partial pressure of CO_2 (see Eqn. S-2). To obtain the parameters used in the model, we fit this equation to the isotherm data for mmen-Mg_2(dobpdc) reported by McDonald *et al.* The adsorbed CO_2 concentration in a direct air capture process would not exceed the equilibrium adsorbed concentration at a partial pressure of 0.4 mbar; therefore, the parameter fitting was restricted for data below this threshold. As shown in Figure S1, this simplified model fit the data of interest fairly well (R^2 =0.964). Relevant parameters were obtained from this fit, the values of which are listed in Table S1.

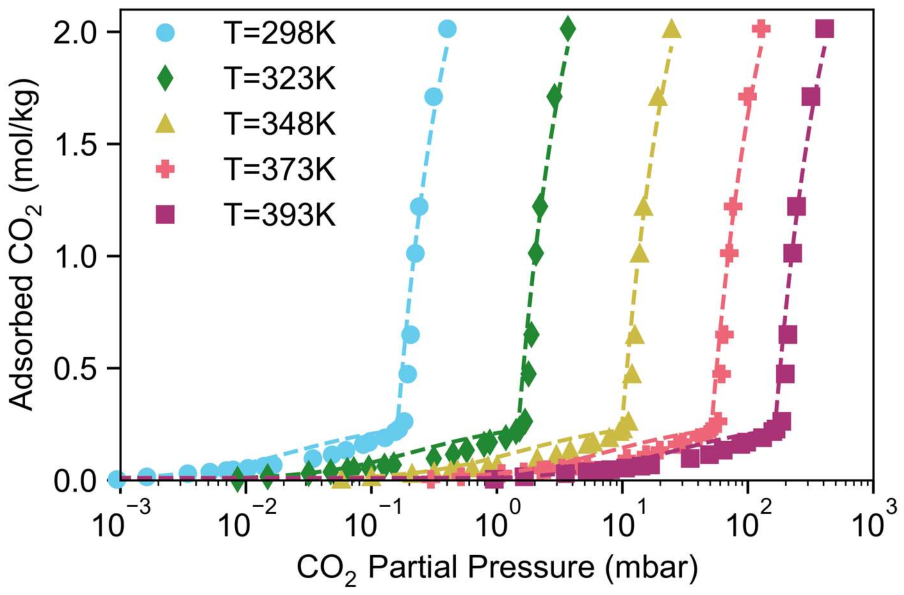

Figure S1. Validation of isotherm model used in this analysis (dotted lines) against literature data (solid symbols).

Table S1: Base-case parameters used in DAC and bioreactor models

Parameter	Value	Unit	Source
DAC Model			
k	0.007	s^{-1}	[7]
Q^{sat1}	0.258	mol/kg	See Note S1
Q^{sat2}	2.791	mol/kg	See Note S1
$p_{\mathrm{step}}(298\mathrm{K})$	0.16	mbar	See Note S1
$K_{\mathrm{eq1}}(298\mathrm{K})$	4.07×10^4	-	See Note S1
$K_{\mathrm{eq2}}(298\mathrm{K})$	6.61×10^3	-	See Note S1
$p_{\mathrm{CO2}}(z = 0)$	0.4	mbar	Assumed
ΔH_{ads}	-71	kJ/mol	[6]
ε	0.95	-	Assumed
L	0.3	m	Assumed
ρ_{ads}	3220	kg/m^3	[6]
τ	0.1	s	Assumed
h_{A}	8.8	$W/m^2 \cdot K$	[8]
r	1	mm	Assumed
T_{s}	120	ºC	Assumed
$c_{\mathrm{p,ads}}$	892.5	$J\ kg^{-1}\ K^{-1}$	[9]
$c_{\mathrm{p,mon}}$	897	$J\ kg^{-1}\ K^{-1}$	www.catalystdpf.com
$m_{\mathrm{mon}}/m_{\mathrm{ads}}$	1.4	-	Assumed
$\hat{c}_{\mathrm{p,CO2}}$	37.2	$J\ mol^{-1}\ K^{-1}$	[10]
p_{vac}	0.1	bar	Assumed
γ	1.3	-	[10]
h_s	18	$W/m^2 \cdot K$	[11]
Knallgas Reactor Model			
$\kappa_{\mathrm{X,H_2,K}}$	5.26		[12]
$\mu_{\mathrm{K,max}}$	0.18	h^{-1}	[12]
$K_{\mathrm{s,H2}}$	20.4	μM	[13]
$K_{s,\mathrm{CO2}}$	9.38	μM	[13]
$K_{s,\mathrm{O2}}$	2.5	μM	[14]
$k_{\mathrm{L}}a_{\mathrm{H2,K}}$	250	h^{-1}	Assumed
H_{H2}	0.00078	$mol\ kg^{-1}\ bar^{-1}$	[10]
H_{CO2}	0.033	$mol\ kg^{-1}\ bar^{-1}$	[10]
H_{O2}	0.0012	$mol\ kg^{-1}\ bar^{-1}$	[10]
T_{K}	30	ºC	DSMZ
$V_{\mathrm{L,K}}/V_{\mathrm{G,K}}$	4	-	Assumed
$D_{\mathrm{gas,K}}$	100	h^{-1}	Assumed
ϕ_{K}	0.2	-	Assumed
$c_{\mathrm{Bu,max,K}}$	10	$g\ L^{-1}$	Assumed
Acetogenic Reactor Model			
$\mu_{\mathrm{max,A,1}}$	0.123	h^{-1}	[15]

Parameter	Value	Unit	Source
$K_{\mathrm{H2,A,1}}$	20	μM	[16]
$K_{\mathrm{CO2,A,1}}$	20	μM	[16]
$c_{\mathrm{Na,min,A,1}}$	0.2	M	[17]
$c_{\mathrm{Na,max,A,1}}$	0.75	M	[18]
$k_{\mathrm{L}}a_{\mathrm{H2,A,1}}$	250	h^{-1}	Assumed
$p_{\mathrm{H2},i,\mathrm{A,1}}$	0.67	atm	Assumed
$p_{\mathrm{CO2},i,\mathrm{A,1}}$	0.33	atm	Assumed
$T_{\mathrm{A,1}}$	35	ºC	DSMZ
$D_{\mathrm{gas,A,1}}$	100	h^{-1}	Assumed
$V_{\mathrm{L,A,1}}/V_{\mathrm{G,A,1}}$	4	-	Assumed
Acetotrophic Reactor Model			
$\kappa_{\mathrm{X,Ac,A,2}}$	1.07		[19]
$\mu_{\mathrm{max,A,2}}$	0.46	h^{-1}	[20]
$K_{\mathrm{Ac,A,2}}$	8.3	mM	[21]
$K_{\mathrm{O2,A,2}}$	2.5	μM	[14]
K_{I}	0.83	M	[22]
$c_{\mathrm{Na,min,A,2}}$	0.2	M	[17]
$c_{\mathrm{Na,max,A,2}}$	1.05	M	[17]
$p_{\mathrm{O2},i,\mathrm{A,2}}$	1	atm	Assumed
$V_{\mathrm{L,A,2}}/V_{\mathrm{G,A,2}}$	4	-	Assumed
$D_{\mathrm{gas,A,2}}$	60	h^{-1}	Assumed
$k_{\mathrm{L}}a_{\mathrm{O_2,A,2}}$	150	h^{-1}	Assumed
$T_{\mathrm{A,2}}$	35	ºC	Assumed
$\phi_{\mathrm{A,2}}$	0.2	-	Assumed
$c_{\mathrm{Bu,max,A,2}}$	10	g L^{-1}	[23]
Process Model			
η_1	0.8	-	Assumed
μ_{air}	1.85×10^{-5}	kg m^{-1} s^{-1}	[24]
η_2	0.8	-	Assumed
P_2	2	atm	Assumed
η_3	0.9	-	Assumed
COP	2.51		[25]
$\Delta H_{\mathrm{comb,H2}}$	286	kJ/mol	[10]
η_4	0.8	-	[26]
$D_{\mathrm{O_2}}$	2.42×10^{-5}	cm^2/s	[11]
$D_{\mathrm{H_2}}$	5.11×10^{-5}	cm^2/s	[11]
$D_{\mathrm{CO_2}}$	1.91×10^{-5}	cm^2/s	[11]
t_{DAC}	2	years	[9]

Table S2: Cost correlations for equipment used in technoeconomic assessment

Equipment	Sizing Variable (Unit)	Correlation Used	Maximum Size	Installation Factor	Source
Heat Pump	Heating Power – P (kW_{th})	$C_{p,HP} = \$1{,}186P^{0.887}$	70,000 kW_{th}	1	[27]
Fan	Air Flow Rate – Q (m^3/s)	$C_{p,fan}/\$ = \exp[7.52 + 0.331 \ln Q + 0.0853(\ln Q)^2]$	200 m^3/s	1.6	[28]
Vacuum Pump	Suction Rate – Q (m^3/s)	$C_{p,vac} = \$169{,}357Q^{0.35}$	0.165 m^3/s	1.6	[28]
DAC Vessel[a]	Volume – V (m^3)	$C_{p,vessel}/\$ = \exp[7.98 + 0.51 \ln V + 0.00582(\ln V)^2]$	12,000 m^3	1.6	[28]
Electrolyzer	Power draw – P (kW)	$C_{p,Elec} = \$900P$	n/a	1.05	[29]
Gas Storage	Volume – V (Nm^3)	$C_{p,GS} = \$79.35V$	n/a	1.6	[30]
Bioreactor	n/a	$C_{p,BR} = \$2{,}341{,}000$	1000 m^3	2.3	[31]
Liquid-Liquid Extractor	Volume – V (m^3)	$C_{p,LLE} = \$8070V^{0.7}$	177 m^3	1.6	[28]
Heat Exchanger	Surface Area – A (m^2)	$C_{p,HX}/\$ = \exp[10.26 - 0.453 \ln A + 0.0979(\ln A)^2]$	1100 m^2	1.6	[28]
Distillation Column		Multiple correlations (following method described by Seider)		1.9	[28]

[a] This correlation assumes that the vessel is a cylinder with a length twice its diameter, and is rated for pressures up to 2 atm.

Table S3: Material costs used in technoeconomic assessment

Material	Unit	Cost/Unit	Source
Sorbent	kg	$63	[9]
Monolithic support	kg	$4.25	www.catalystdpf.com
Nitrogen gas	kg	$0.76	www.chemanalyst.com
Process water	1000 gal	$4.70	[32]
Ammonia	kg	$1.18	www.chemanalyst.com
Phosphoric acid	kg	$1.30	www.chemanalyst.com
Magnesium sulfate	kg	$0.38	www.chemanalyst.com
Sodium hydroxide	kg	$0.63	www.chemanalyst.com
Sulfuric acid	kg	$0.26	www.chemanalyst.com
Mesitylene	kg	$4.04	[33]
Biosolids waste disposal	kg dry	$0.33	[34]
Organic waste disposal	kg COD	$0.16	[35]
Reverse osmosis	kmol solute	$1.38	[36]

Table S4: Capital cost contributions and their method of calculation (based on TEA of cellulosic biofuel plant described by Davis *et al.*)[37]

Capital Cost Contributions	Cost Calculation
Installed Equipment Cost (IEC)[a]	$\mathrm{IEC} = \sum n_i F_i C_i$
Warehouse Costs	0.04×IEC
Site Development	0.09×IEC
Additional Piping	0.045×IEC
**Total Direct Costs (TDC)**	Sum of above
Prorateable Costs	0.1×TDC
Field Expenses	0.1×TDC
Home Office and Construction	0.2×TDC
Project Contingency	0.1×TDC
Other Indirect Costs	0.1×TDC
**Total Indirect Costs (TIC)**	Sum of above
**Fixed Capital Investment (FCI)**	TDC+TIC
Working Capital (WC)	0.05×FCI
**Total Capital Investment (TCI)**	FCI+WC

[a] n_i is the number of units required, F_i is the installation factor, and C_i is the unit cost of equipment piece i.

Table S5: Operating cost contributions and their method of calculation

Operating Cost Contributions	Cost Calculation
**Variable Operating Costs (VOC)** [a]	$\mathrm{VOC} = \sum \dot{m}_j c_j t_{\mathrm{op}}$
Labor Costs (LC)	\$7.4 MM/yr [b]
Labor Burden [37]	0.9×LC
Maintenance Costs [37]	0.03×IEC
Property Insurance [37]	0.007×FCI
**Fixed Operating Costs (FOC)**	Sum of above
**Total Operating Costs (TOC)**	VOC+FOC

[a] $\dot{m}_j$ refers to the demand of material (or energy) *j* per unit time, c_j is the unit cost of material (or energy) *j*, and t_{op} is the time of operation. For the annual variable operating cost, t_{op} is 7920 h (330-day uptime, 24 hours per day).

[b] Due to the higher complexity of the processing components involved, we assume that the process described here would require roughly double the total labor costs of a cellulosic ethanol plant operating at the same scale described in the TEA by Humbird *et al.*[38] This value is also adjusted for inflation and regional variation (BLS Employment Cost Index), leading to an annual employee salaries cost of \$7.4 million per year.

Table S6: Discounted cash flow rate of return analysis parameters

**Parameter**	**Value**
Plant Life	30 Years
Discount Rate [a]	10%
Federal Income Tax Rate [b]	21%
Plant Depreciation	200% Declining Balance
Plant Recovery Period [c]	5 Years
Equity Financing	100%
Construction Period [d]	24 months

[a] A 10% discount rate is assumed, based on the recommendation by the U.S. Department of Energy for renewable energy technologies.[39]
[b] A tax rate of 21%, consistent with the federal corporate tax rate in the United States, is assumed.
[c] The IRS Modified Accelerated Cost Recovery System (MACRS) is used to determine plant depreciation, based on the Asset Class 28.0 "Manufacture of Chemical and Allied Products", which uses a GDS recovery period of 5 years.[40]
[d] A construction period of 24 months, with capital expended evenly over the period, followed by a 30-year plant lifetime, with 100% production capacity beginning immediately following construction, is assumed.

Note S5: Optimizing DAC cycling conditions for maximum productivity

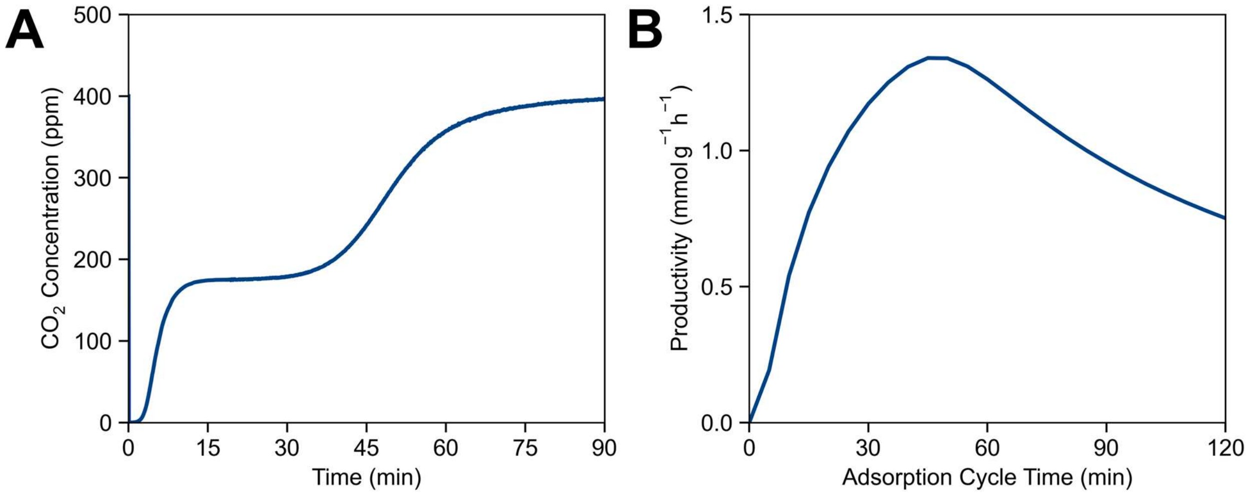

Figure S2. (A) Simulated breakthrough curve of CO_2 adsorption under base-case operating conditions. (B) Effect of length of adsorption phase on the overall productivity of carbon capture.

Model parameters were set to their base-case values as listed in Table S1, and adsorption by the MOF sorbent was simulated (Fig. S2A). Two distinct phases of adsorption are observed, due to the step behavior originally observed by McDonald *et al.*[6] This behavior is in agreement with the results of the MOF model presented by Sinha *et al.* [9]

As expected, the fastest rate of adsorption occurs at the beginning of the adsorption process and slows as the number of available chemisorption sites decreases. Therefore, utilizing the entire MOF capacity is likely not the most efficient use of time when running a DAC process. To study this optimization problem, we simulated the adsorption and desorption cycles with a variety of assumed adsorption cycle times, varying from 0 to 120 min (Fig. S2B). We assume that the desorption cycle time is fixed (15 min) and that there is a 3 min deadtime due to the process switching from adsorption to desorption mode. As the adsorption cycle time increases, the quantity of CO_2 adsorbed per cycle increases. A carbon capture productivity is then

calculated by dividing this quantity by the entire time taken to complete a carbon capture cycle (adsorption, desorption, and deadtime). At low values of the adsorption cycle time, the desorption and deadtime make up a relatively large fraction of the overall cycle time and therefore drive the productivity down. However, with very long adsorption times, the average rate of carbon capture decreases as the driving force for CO_2 capture falls dramatically as the sorbent nears its capacity. Under base-case operating parameters, an optimum is found for an adsorption cycle time of 50 min, corresponding to a productivity of 1.34 mol CO_2 kg^{-1} sorbent h^{-1} with 1.52 mol kg^{-1} captured each cycle. These conditions will be assumed in the overall process simulation for the rest of the study.

Note S6: Optimizing bioprocess productivity

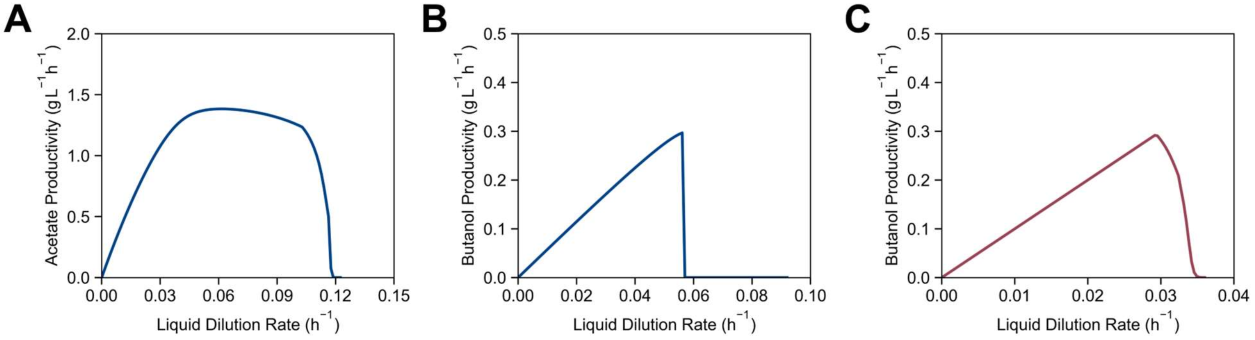

Figure S3. Effect of bioreactor dilution rate on productivity. (A) Volumetric production rate of acetate in the first bioreactor in the acetogen-based system under base-case operating conditions as a function of the dilution rate. **(B)** Volumetric production rate of butanol in the second bioreactor in the acetogen-based system under base-case operating conditions as a function of the dilution rate. **(C)** Volumetric production rate of butanol in the Knallgas bacteria-based system under base-case operating conditions as a function of the dilution rate.

Volumetric productivity is the key metric to be optimized in each of the three bioreactors (both bioreactors in the acetogen-based system, and the bioreactor in the Knallgas bacteria-based system). For each bioreactor, for a given set of parameters and operating conditions, there will be a dilution rate that maximizes productivity in the bioreactor. An example for each bioreactor is shown in Fig. S3, which shows the effect that the liquid dilution rate has on the productivity of each bioreactor, under base-case assumptions.

For the first bioreactor in the acetogen-based system, which converts CO_2 and H_2 to acetate, the volumetric rate of acetate production is the value to optimize (Fig. S3A). A general trend is seen, in which productivity initially rises with dilution rate, as higher dilution rates enable faster specific growth rates at steady-state and therefore higher acetate production rates. However, as the liquid dilution rate exceeds its optimal value, effects of salinity begin to limit acetate production. Growth rates that are high enough to avoid cell washout can only occur if the concentration of sodium (which is added in the form of NaOH in stoichiometric proportions to the acetic acid) is sufficiently low. Therefore, as dilution rate continues to increase, the acetate titer becomes lower, leading to a decrease in productivity. Total cell washout occurs when the dilution rate approaches the maximum specific growth rate of the acetogen (0.123 h^{-1}).

The second bioreactor in the acetogen-based system, which contains an acetotroph that converts acetate to n-butanol, will similarly have a dilution rate that maximizes the productivity of the bioreactor. In this case, the volumetric butanol production rate is the key metric to optimize. In this reactor, the productivity is limited by the rate at which the substrate (acetate) is fed to the bioreactor. Therefore, the butanol productivity increases roughly linearly with increasing dilution rates (Fig. S3B). This occurs until the dilution rate exceeds the maximum specific growth rate achievable under these conditions, at which point cell washout occurs rapidly.

Butanol productivity is also optimized in the Knallgas bacteria-based bioprocess. Two regimes are observed when simulating this chemostat at various dilution rates. Under low dilution rates, biomass and butanol will accumulate in the bioreactor until the maximum tolerated butanol concentration is reached (Fig. S3C). The productivity is simply equal to the maximum butanol concentration multiplied by the dilution rate. Therefore, this regime is specifically limited by the butanol tolerance of the microbe. At high dilution rates, butanol will not accumulate to the tolerance limit, and therefore productivity is not limited by butanol toxicity. Rather, the productivity is limited by the gas-liquid mass transfer rate of the gaseous substrates into the medium. In this gas-liquid mass transfer-limited regime, butanol productivity will decrease with increasing dilution rate until cell washout occurs (D=0.036 h^{-1} under base-case conditions). The maximum productivity is observed at the intersection between the butanol toxicity- and mass transfer-limited regimes.

Note S7: Impact of butanol toxicity on Knallgas bacteria-based system

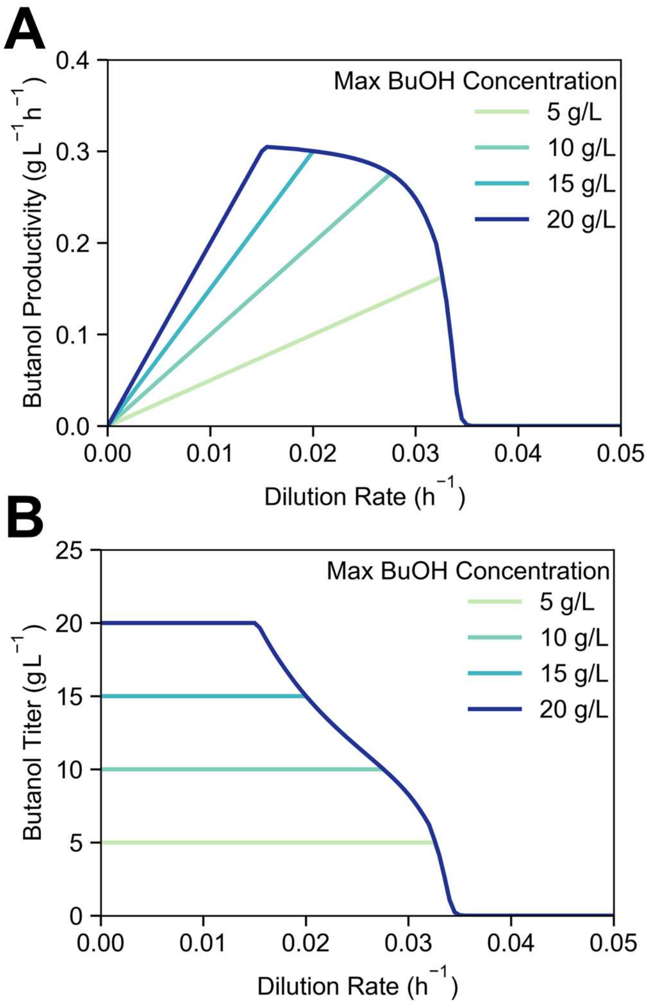

Figure S4 Effect of butanol tolerance on the productivity (A) and titer (B) in the Knallgas bacteria-based EMP system.

As mentioned in the main text, the performance of the Knallgas bacteria-based system is heavily dependent on the butanol tolerance of the microbe. In our model, we define a simple parameter ($c_{\mathrm{Bu,max,K}}$) that essentially limits the possible titer. Curves describing the effect of dilution rate on butanol productivity at various values of the parameter $c_{\mathrm{Bu,max,K}}$ are shown in Fig. S4A. As described in Note S6, there are two regimes observed in the graph depicting the effect of dilution rate on productivity: a gas-liquid mass transfer-

limited regime and a butanol-toxicity-limited regime. As the maximum tolerated butanol concentration increases, the gas-liquid mass transfer-limited regime is encountered at lower dilution rates, as butanol toxicity becomes less of a determinant of the bioreactor productivity. Therefore, even if the effect of butanol toxicity is completely removed (*i.e.*, $c_{\mathrm{Bu,max,K}}$ becomes infinite), the productivity will still be limited by gas-liquid mass transfer.

The butanol titer is even more dependent on the maximum butanol concentration tolerated (Fig. S4B). In the butanol toxicity-limiting regime, the butanol titer is equal to the parameter $c_{\mathrm{Bu,max,K}}$. In the gas-liquid mass transfer-limited regime, the system is not limited by butanol toxicity effects, and therefore the butanol titer will be below the maximum butanol concentration tolerated.

Clearly, the butanol tolerance of the microbe is an important parameter when modeling the performance of the Knallgas bacteria-based bioprocess. As productivity and titer are both major cost drivers (see Fig. 4 in the main text), this parameter will be important to the economics of the process. There are limited data in the literature on the tolerance of Knallgas bacteria to n-butanol, and therefore this key parameter is subject to moderate uncertainty. For context, wild-type *E. coli* can tolerate n-butanol up to ~1% v/v (8.1 g L^{-1}), but the limit has been improved to ~1.6% v/v (13 g L^{-1}) through various microbial engineering strategies.[23,41] *Clostridium acetobutylicum*, a natural butanol producer known for its use in ABE fermentations, normally tolerates butanol up to 2% v/v (16 g L^{-1}), but with improvements can tolerate up to 3% v/v (24 g L^{-1}).[42] Even if the native butanol tolerance of Knallgas bacteria is low, the use of adaptive laboratory evolution to improve solvent tolerance of microbes is usually successful;[43] therefore a butanol tolerance of 10 g L^{-1} is a reasonable base-case assumption.

Note S8: Effect of gas recycle on fermentation processes

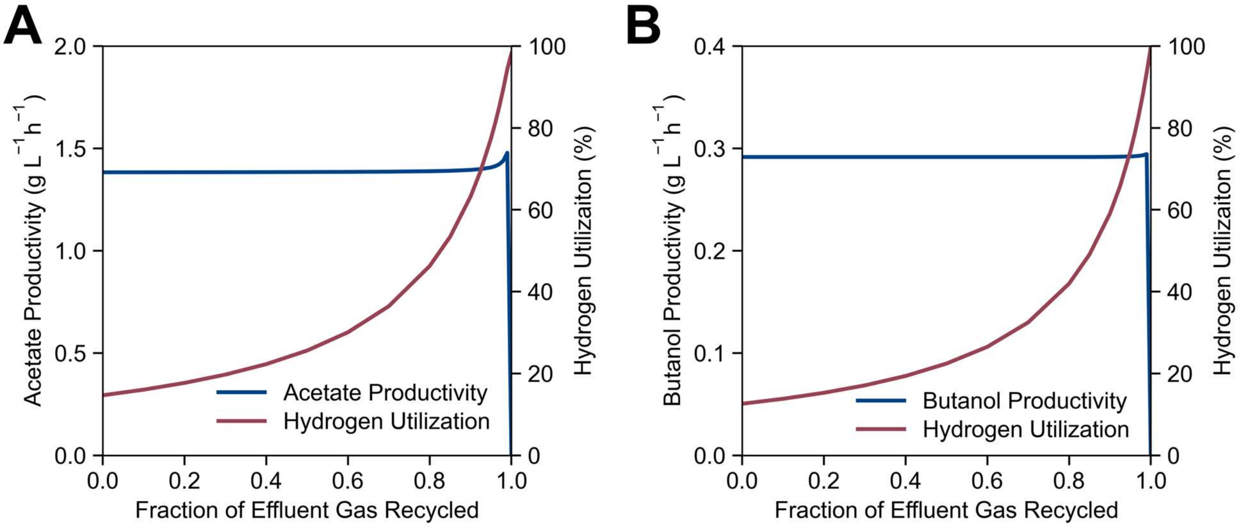

Figure S5. Effect of gas recycle on the productivity of **(A)** acetate generation in the first bioreactor of the acetogen-based process and **(B)** butanol generation in the Knallgas bacteria-based process.

The effect of gas recycle on the performance of the two gas fermentation processes was explored. The gaseous substrates fed to the bioreactors are quite costly, and therefore maximizing the utilization of each substrate is critical for an economical bioprocess. Fortunately, the substrates are fed in the gas phase, and unused substrates can be recycled with relative ease. However, recycling all gases exhausted from the reactor can pose issues due to the accumulation of inert compounds. In base-case DAC simulations, the purity of the captured CO_2 is calculated to be 98.3%, with the balance being N_2. Whether this impurity will substantially affect the ability to recycle hydrogen and carbon dioxide is studied.

The model is slightly modified such that the feed gas stream is a mixture of fresh gas and some fraction of the gas effluent. Assuming that CO_2 is provided from the DAC process with a purity of 98.3%, the effect of the gas recycle fraction (the fraction of the effluent gas stream that is recycled rather than purged) on the bioprocess performance is then evaluated (Fig. S5). As expected, the percent of H_2 utilized approaches 100% as the gas stream is completely recycled. However, in both systems, productivity does decline rapidly when the recycle fraction exceeds 99%, as the inert N_2 is accumulated to such a degree that the H_2 and CO_2 concentrations in the feed stream become too low. Therefore, the recycle fraction is restricted to this value or lower. This still allows significant recycling of the gases, with hydrogen utilization around 97%, indicating that nearly all of the produced hydrogen is reacted. Based on these results, the recyclability and by extension the utilization of the gaseous substrates should not be an issue.

Note S9: Economic modeling of waste disposal and treatment

Throughout the process, numerous byproducts and waste streams are generated. As described by the bioprocess model equations, bacterial biomass is generated concurrently with butanol in both process options. The solid cell biomass must be separated from the medium following fermentation and prior to the liquid-liquid extraction step. We assume that the separation of cells from the medium can be achieved by low-energy, low-cost methods (*e.g.*, settling) and therefore will not be considered as a major unit operation in our cost model. The cell biomass is then disposed of as sludge at a fixed disposal cost. Peccia and Westerhoff gave a reasonable range of $100-800 per dry ton of sludge, depending on the method of disposal, in 2015.[34] By selecting a value of $250/ton in 2015$ as a base-case value, we adjust for inflation to a value of $334/ton in 2022.

To reduce the water footprint of the process, water is recycled. The raffinate stream coming from the liquid-liquid extraction process can be recirculated to the fermenters. This stream, however, must be treated such that the impurities accumulated in this stream do not detrimentally affect the bioprocess. In bioprocesses, contaminants may come from unconsumed substrates, organic compounds produced as unwanted side-products during the fermentation, leftover products that are not recovered, and salts added during the process.[44] We assume unconsumed substrates, organic byproducts, and leftover butanol can be removed by standard (aerobic/anaerobic) wastewater treatment systems, with a fixed cost of $0.16/ kg chemical oxygen demand (COD), equivalent to that of wastewater treatment for a 1st generation biorefinery according to Li *et al.*[35] The chemical oxygen demand of the waste stream is calculated by the stoichiometry of combustion of each species.

The unconsumed acetate is explicitly modeled (Eq. S-49), while the substrates in the Knallgas bacteria bioreactor are gaseous and therefore do not need to be removed during the water treatment step. We assume organic byproducts are generated during butanol production, based on the results provided by Saini *et al.*, in fixed proportions to the amount of biomass formed,[45] which translates to 0.945 kg COD per kg of biomass produced. The amount of butanol in the stream that is not extracted during the separations process is calculated from the separations model.

The salt generated from pH control can be removed by reverse osmosis downstream of the aerobic/anaerobic wastewater treatment systems. We assume that the cost of salt removal is equivalent to the cost of seawater desalination on a molar basis of ions removed. Based on the data provided by Quon *et al.*, we calculate this to be $1.38 per kmol of salt removed (in 2022$).[36] The Knallgas system does not require desalting, as pH control is not required according to the bioprocess model.

Table S7A: Equipment sizing and estimated costs for acetogen-based system (base-case scenario)

Equipment	Unit Size	Unit Cost	Number Of Units	Installed Equipment Cost
DAC Heat Pump	52.1 MW	$18.1MM	3	$54.3 MM
Fans	200 m^3/s	$120K	412	$77.3 MM
Vacuum Pump	0.165 m^3/s	$90K	152	$21.9 MM
DAC Vessel	12,000 m^3	$590K	1	$0.9 MM
Electrolyzer [a]	1 MW	$900K	494	$467.1 MM
Gas Storage Tanks	1,000 Nm^3	$79K	271	$34.4 MM
Bioreactor 1	1,000 m^3	$2.3MM	48	$258.5 MM
Bioreactor 2	1,000 m^3	$2.3MM	55	$296.1 MM
Liquid-Liquid Extractor	174 m^3	$300K	26	$12.4 MM
Distillation Columns	[b]	$1.2MM	15	$34.9 MM
Separations Heat Pump	57.4 MW	$19.7MM	3	$59.2 MM
Heat Exchanger	1080 m^2	$140K	39	$8.9 MM
Total IEC				$1326 MM

Table S7B: Equipment sizing and estimated costs for Knallgas bacteria-based system (base-case scenario)

Equipment	Unit Size	Unit Cost	Number Of Units	Installed Equipment Cost
DAC Heat Pump	35.7 MW	$13.0 MM	2	$25.9 MM
Fans	200 m^3/s	$120K	189	$35.3 MM
Vacuum Pump	0.165 m^3/s	$90K	70	$10.1 MM
DAC Vessel	5,500 m^3	$360K	1	$0.6 MM
Electrolyzer [a]	1 MW	$900K	509	$481.4 MM
Gas Storage Tanks	1,000 Nm^3	$79K	229	$29.0 MM
Bioreactor	1,000 m^3	$2.3MM	55	$296.1 MM
Liquid-Liquid Extractor	173 m^3	$300K	14	$6.7 MM
Distillation Columns	[b]	$1.2MM	9	$19.4 MM
Separations Heat Pump	47.6 MW	$16.7MM	2	$33.4 MM
Heat Exchanger	1080 m^2	$140K	21	$4.8 MM
Total IEC				$943 MM

[a] An electrolyzer capital cost of $900,000/MW is assumed independent of scale. The effects of the expected reduction in hydrogen production costs are explored in Fig. 3 of the main text.
[b] Distillation columns are 5 m in diameter and 14 m high, with 20 stages.

Table S8: Economic summary of DAC-EMP process under base-case assumptions

	**Acetogen**	**Knallgas**
Installed Equipment Cost (IEC)	$1.33 BB	$943 MM
**Total Capital Investment**	**$2.62 BB**	**$1.86 BB**
Variable Operating Costs	$780 MM/y	$331 MM/y
Fixed Operating Costs	$71 MM/y	$55 MM/y
**Total Operating Costs**	**$851 MM/y**	**$386 MM/y**
Butanol Production	40 MM gal/y	40 MM gal/y
Revenue	$1.17 BB/y	$613 MM/y
**Minimum Selling Price**	**$29.27**	**$15.33**

Note S10: Assumptions regarding parameter sensitivity analysis and interpretation of Table S9

The subsection "Parameter sensitivity analysis" in the results section in the main text describes the purpose of the sensitivity analysis. In all, 38 parameters were selected for analysis. Details regarding the selection of the baseline value and the range of values tested are described here.

The base-case scenario assumes a plant lifetime of 30 years, which is the standard for similar TEAs,[38] and a range of plant lifetimes from 10 to 50 are considered in the sensitivity study. Deviations of ± 50% are applied to the factor relating the installed equipment cost and the fixed capital investment (CAPEX/IEC). Production scales are considered that are an order or magnitude lower and higher than the baseline scale, as effects on the price are unlikely to be observed for smaller deviations. A discount rate of 10% is the baseline discount rate (see Table S6), and a range from 6%-14% is chosen somewhat arbitrarily.

A range of values for the coefficient of performance is provided by Zuberi *et al*. while the baseline value is taken from Deutz and Bardow.[25,27] The values for equipment efficiency range from 0.6 – 1.0, with the upper value representing a scenario in which no inefficiency occurs for that piece of equipment.

Growth rates for the Knallgas bacteria range from 0.1 to 0.42, depending on oxygen partial pressure, according to Ishizaki and Tanaka, with the base case value (0.18 h^{-1}) being the one that most closely corresponds with our assumed O_2 partial pressure (0.16 atm).[12] The range of reasonable k_La's for both hydrogen and oxygen is based on the ranges observed by Vasconcelos *et al*. for six-bladed disk turbine impellers.[4] Carbon selectivity values are chosen arbitrarily, as they will depend on the results of metabolic engineering and other factors. Maximum butanol concentration tolerated by the strains range from 5 to 25 g L^{-1}, roughly in line with empirical ranges for a variety of bacteria described in Note S7. Maximum tolerated sodium concentrations range from 0.25 M to 1.25 M. 1.0 M is a typical concentration of sodium that can be tolerated by non-halophilic strains such as *E. coli*,[17] while the upper range represents an additional 25% to examine the effect of hypothetical improvements that could be attained by strain engineering. The range for acetogen growth rates is 0.02 to 0.15 h^{-1}. Growth rates in the 0.02 to 0.12 h^{-1} range were observed for different acetogens by Groher and Weuster-Botz,[15] and the upper bound also represents a hypothetical 25% improvement. The values for the growth rate of acetotrophs are 0.2 to 0.58 h^{-1}. The baseline value (0.46 h^{-1}) is the growth rate for *E. coli* on acetate observed by Noh *et al.*; the lower bound represents the lowest growth rate of *E. coli* in defined medium observed by Andersen and Meyenburg, and the upper bound represents a hypothetical 25% improvement compared to the base case value.[19,20] Excluded from the parameter sensitivity analysis are physical parameters (*e.g.*, diffusivities, Henry's Law constants) and operating conditions that are considered "fixed" for the process (*e.g.*, operating temperatures and pressures).

The installed cost of each equipment item, as well as the unit cost of all materials, are varied by ± 50% from their baseline value to represent a reasonable range for both the uncertainty in equipment cost estimates as well as the fluctuations of material prices. An exception to this is the electrolyzer cost, which is varied in

even intervals from \$100/kW to \$900/kW, representing the optimistic and basecase values described in Fig. 3 in the main text. Another exception is for the price of electricity, which is varied from \$0.00 to \$0.04/kWh in even intervals, as it is already established that electricity prices must be very low for EMP to be industrially relevant. Excluded from the sensitivity analysis are the cost of sorbent, NaOH, and H_2SO_4, as the sensitivity of each process to those costs has already been detailed in Fig. 3.

Each parameter is varied to five distinct values in the chosen range (labeled P1 through P5), and the cost of butanol is recalculated for each of those values. The baseline value of each parameter is bolded in Table S9. Some values (*e.g.*, Knallgas bacteria growth rate) only affect the economics of one system. In such cases, butanol selling prices are still listed for the other system yet are unchanged from the baseline.

Table S9: Results of parameter sensitivity analysis

	Parameter Values					Butanol Price - Knallgas System ($/gal)					Butanol Price - Acetogen System ($/gal)				
**Parameter (units)**	**P1**	**P2**	**P3**	**P4**	**P5**	**P1**	**P2**	**P3**	**P4**	**P5**	**P1**	**P2**	**P3**	**P4**	**P5**
**Financial Model**															
Dicsount Rate	6%	8%	**10%**	12%	14%	$ 7.24	$ 7.75	**$ 8.30**	$ 8.87	$ 9.48	$ 11.02	$ 11.92	**$ 12.88**	$ 13.90	$ 14.97
Plant Life (y)	10	20	**30**	40	50	$ 9.79	$ 8.60	**$ 8.30**	$ 8.19	$ 8.16	$ 15.53	$ 13.42	**$ 12.88**	$ 12.70	$ 12.64
CAPEX/IEC	1.44	1.66	**1.88**	2.1	2.32	$ 7.53	$ 7.91	**$ 8.30**	$ 8.68	$ 9.06	$ 11.53	$ 12.20	**$ 12.88**	$ 13.56	$ 14.24
Scale (gal/y)	4,000,000	10,000,000	**40,000,000**	100,000,000	400,000,000	$ 8.67	$ 8.40	**$ 8.30**	$ 8.27	$ 8.26	$ 13.36	$ 12.97	**$ 12.88**	$ 12.84	$ 12.82
**Process Model**															
COP	1.6	2	**2.51**	4	5.8	$ 8.45	$ 8.37	**$ 8.30**	$ 8.19	$ 8.14	$ 13.21	$ 13.03	**$ 12.88**	$ 12.67	$ 12.56
Blower efficiency	0.6	0.7	**0.8**	0.9	1	$ 8.30	$ 8.30	**$ 8.30**	$ 8.29	$ 8.29	$ 12.90	$ 12.89	**$ 12.88**	$ 12.88	$ 12.87
Vacuum pump efficiency	0.6	0.7	**0.8**	0.9	1	$ 8.30	$ 8.30	**$ 8.30**	$ 8.29	$ 8.29	$ 12.90	$ 12.89	**$ 12.88**	$ 12.88	$ 12.87
Heat loss efficiency	0.6	0.7	0.8	**0.9**	1	$ 8.43	$ 8.37	$ 8.33	**$ 8.30**	$ 8.25	$ 13.19	$ 13.05	$ 12.96	**$ 12.88**	$ 12.82
Electrolyzer efficiency	0.6	0.7	**0.8**	0.9	1	$ 9.09	$ 8.64	**$ 8.30**	$ 8.03	$ 7.82	$ 13.66	$ 13.21	**$ 12.88**	$ 12.62	$ 12.42
**Bioprocess Model**															
Knallgas growth rate (h^{-1})	0.1	**0.18**	0.26	0.34	0.42	$ 9.77	**$ 8.30**	$ 7.97	$ 7.88	$ 7.85	$ 12.88	**$ 12.88**	$ 12.88	$ 12.88	$ 12.88
Hydrogen k_La (h^{-1})	100	175	**250**	325	400	$ 10.13	$ 8.70	**$ 8.30**	$ 8.18	$ 8.16	$ 18.66	$ 13.47	**$ 12.88**	$ 12.94	$ 12.96
Carbon selectivity	0.2	0.5	**0.8**	0.9	0.95	$ 38.43	$ 13.92	**$ 8.30**	$ 9.21	$ 13.03	$ 57.05	$ 20.59	**$ 12.88**	$ 13.16	$ 15.70
Max BuOH Conc. (g/L)	5	**10**	15	20	25	$ 11.30	**$ 8.30**	$ 7.53	$ 7.24	$ 7.07	$ 13.86	**$ 12.88**	$ 12.88	$ 12.88	$ 12.88
Acetogen growth rate (h^{-1})	0.02	0.05	0.08	**0.12**	0.15	$ 8.30	$ 8.30	$ 8.30	**$ 8.30**	$ 8.30	$ 20.60	$ 14.99	$ 13.56	**$ 12.88**	$ 12.84
Max sodium Conc. (M)	0.25	0.5	**0.75**	1	1.25	$ 8.30	$ 8.30	**$ 8.30**	$ 8.30	$ 8.30	$ 27.08	$ 16.18	**$ 12.88**	$ 11.77	$ 11.49
Acetotroph growth rate (h^{-1})	0.2	0.28	0.37	**0.46**	0.58	$ 8.30	$ 8.30	$ 8.30	**$ 8.30**	$ 8.30	$ 15.66	$ 14.25	$ 13.39	**$ 12.88**	$ 12.45
Oxygen k_La, acetotroph (h^{-1})	100	**150**	250	325	400	$ 8.30	**$ 8.30**	$ 8.30	$ 8.30	$ 8.30	$ 12.86	**$ 12.88**	$ 12.93	$ 12.98	$ 13.03
**Equipment costs**															
Heat Pump cost	-50%	-25%	**0%**	25%	50%	$ 8.08	$ 8.19	**$ 8.30**	$ 8.40	$ 8.51	$ 12.48	$ 12.68	**$ 12.88**	$ 13.08	$ 13.29
Fan cost	-50%	-25%	**0%**	25%	50%	$ 8.17	$ 8.23	**$ 8.30**	$ 8.36	$ 8.42	$ 12.61	$ 12.74	**$ 12.88**	$ 13.02	$ 13.16
Vacuum pump cost	-50%	-25%	**0%**	25%	50%	$ 8.26	$ 8.28	**$ 8.30**	$ 8.31	$ 8.33	$ 12.80	$ 12.84	**$ 12.88**	$ 12.92	$ 12.96
DAC Vessel cost	-50%	-25%	**0%**	25%	50%	$ 8.29	$ 8.29	**$ 8.30**	$ 8.30	$ 8.30	$ 12.88	$ 12.88	**$ 12.88**	$ 12.88	$ 12.89
Gas storage cost	-50%	-25%	**0%**	25%	50%	$ 8.19	$ 8.24	**$ 8.30**	$ 8.35	$ 8.40	$ 12.76	$ 12.82	**$ 12.88**	$ 12.94	$ 13.00
Bioreactor cost	-50%	-25%	**0%**	25%	50%	$ 7.24	$ 7.77	**$ 8.30**	$ 8.82	$ 9.35	$ 10.91	$ 11.90	**$ 12.88**	$ 13.87	$ 14.85
Liquid-liquid extractor cost	-50%	-25%	**0%**	25%	50%	$ 8.27	$ 8.28	**$ 8.30**	$ 8.31	$ 8.32	$ 12.84	$ 12.86	**$ 12.88**	$ 12.90	$ 12.93
Heat Exchanger cost	-50%	-25%	**0%**	25%	50%	$ 8.28	$ 8.29	**$ 8.30**	$ 8.30	$ 8.31	$ 12.85	$ 12.87	**$ 12.88**	$ 12.90	$ 12.91
Distillation column cost	-50%	-25%	**0%**	25%	50%	$ 8.23	$ 8.26	**$ 8.30**	$ 8.33	$ 8.36	$ 12.76	$ 12.82	**$ 12.88**	$ 12.94	$ 13.01
Electrolyzer cost	**100**	300	500	700	900	**$ 8.30**	$ 9.06	$ 9.82	$ 10.58	$ 11.34	**$ 12.88**	$ 13.62	$ 14.36	$ 15.10	$ 15.84
**Material costs**															
Electricity cost	$0.00	$0.01	**$0.02**	$0.03	$0.04	$ 5.93	$ 7.11	**$ 8.30**	$ 9.49	$ 10.67	$ 10.18	$ 11.53	**$ 12.88**	$ 14.23	$ 15.58
Monolith cost	-50%	-25%	**0%**	25%	50%	$ 8.29	$ 8.30	**$ 8.30**	$ 8.30	$ 8.31	$ 12.87	$ 12.87	**$ 12.88**	$ 12.89	$ 12.89
Nitrogen cost	-50%	-25%	**0%**	25%	50%	$ 8.26	$ 8.28	**$ 8.30**	$ 8.32	$ 8.34	$ 12.80	$ 12.84	**$ 12.88**	$ 12.92	$ 12.96
Process Water cost	-50%	-25%	**0%**	25%	50%	$ 8.29	$ 8.29	**$ 8.30**	$ 8.31	$ 8.31	$ 12.87	$ 12.87	**$ 12.88**	$ 12.89	$ 12.89
Ammonia cost	-50%	-25%	**0%**	25%	50%	$ 8.20	$ 8.25	**$ 8.30**	$ 8.35	$ 8.40	$ 12.72	$ 12.80	**$ 12.88**	$ 12.96	$ 13.04
Phosphoric acid cost	-50%	-25%	**0%**	25%	50%	$ 8.25	$ 8.27	**$ 8.30**	$ 8.33	$ 8.35	$ 12.79	$ 12.84	**$ 12.88**	$ 12.92	$ 12.97
Magnesium sulfate cost	-50%	-25%	**0%**	25%	50%	$ 8.29	$ 8.29	**$ 8.30**	$ 8.31	$ 8.31	$ 12.85	$ 12.87	**$ 12.88**	$ 12.89	$ 12.91
Mesitylene cost	-50%	-25%	**0%**	25%	50%	$ 7.95	$ 8.12	**$ 8.30**	$ 8.48	$ 8.65	$ 12.29	$ 12.58	**$ 12.88**	$ 13.18	$ 13.47
Biosolids disposal cost	-50%	-25%	**0%**	25%	50%	$ 8.12	$ 8.21	**$ 8.30**	$ 8.39	$ 8.48	$ 12.60	$ 12.74	**$ 12.88**	$ 13.02	$ 13.16
Organic waste disposal cost	-50%	-25%	**0%**	25%	50%	$ 8.21	$ 8.26	**$ 8.30**	$ 8.34	$ 8.39	$ 12.70	$ 12.79	**$ 12.88**	$ 12.97	$ 13.06
Reverse osmosis cost	-50%	-25%	**0%**	25%	50%	$ 8.30	$ 8.30	**$ 8.30**	$ 8.30	$ 8.30	$ 12.66	$ 12.77	**$ 12.88**	$ 12.99	$ 13.10

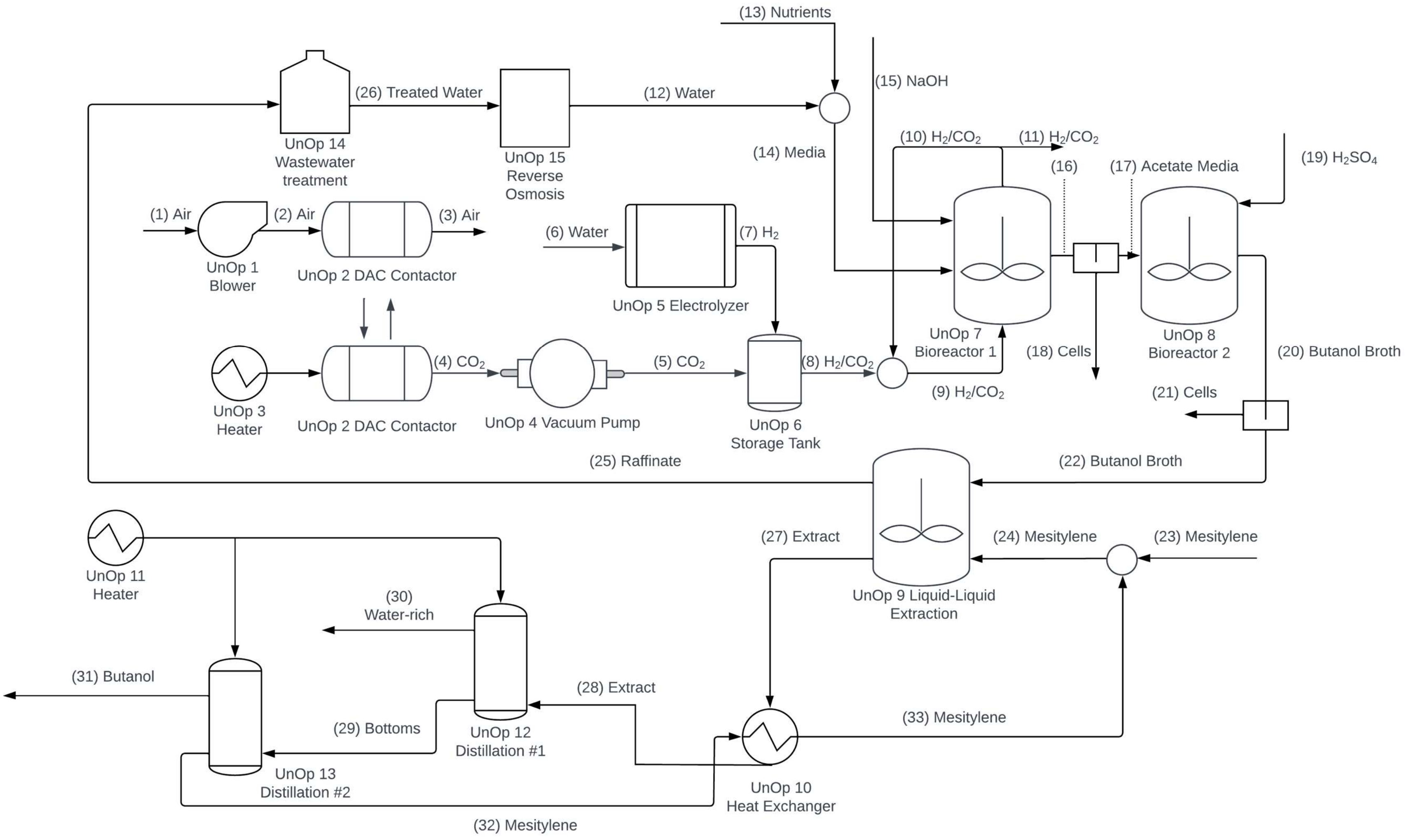

Figure S6. Detailed process flow diagram for acetogen-based DAC-EMP process

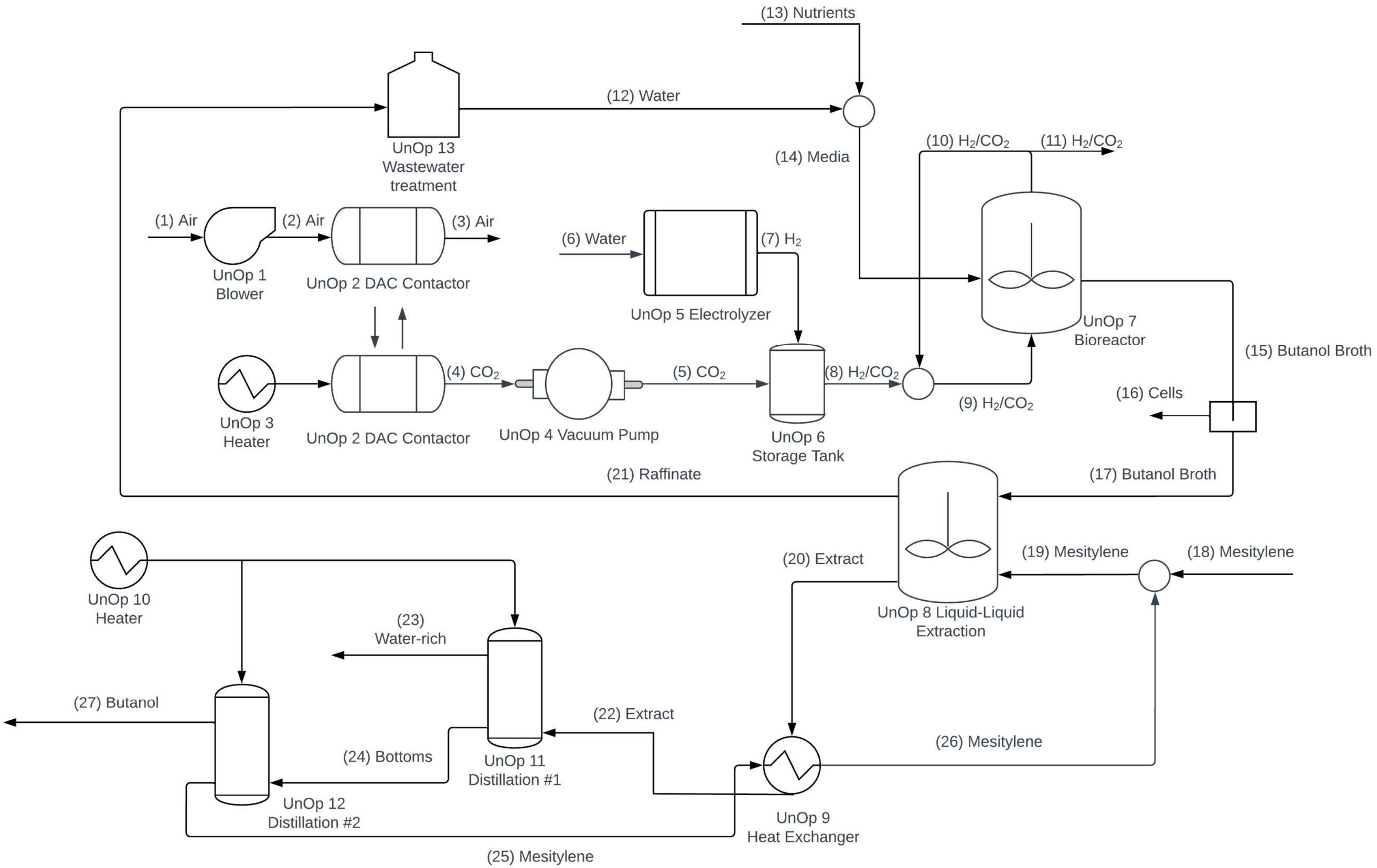

Figure S7. Detailed process flow diagram for Knallgas bacteria-based DAC-EMP process

Table S10: Base-case material flows for acetogen-based DAC-EMP process (stream labels shown in Figure S6)

Stream No.	Components	Flow Rates (kg/h)	Properties [a]	Stream No.	Components	Flow Rates (kg/h)	Properties
**1**	Air	352,000,000		**16**	H_2O	3,010,000	T = 35 °C
	CO_2	214,000			Sodium acetate	93,500	
**2**	Air	352,000,000	P = 133 Pa		Biomass	3,140	
	CO_2	214,000	(guage)		NH_3	891	
**3**	Air	352,000,000			H_3PO_4	428	
	CO_2	107,000			$MgSO_4$	460	
**4**	CO_2	106,000	T= 120 °C	**17**	H_2O	3,010,000	T = 35 °C
	N_2	1,080	P = 0.1 atm		Sodium acetate	93,500	
**5**	CO_2	106,000	T = 120 °C		NH_3	891	
	N_2	1,080	P = 2 atm		H_3PO_4	428	
**6**	H_2O	89,700			$MgSO_4$	460	
**7**	H_2	9,960		**18**	Biomass	3,140	
**8**	H_2	9,960		**19**	H_2SO_4	51,600	
	CO_2	106,000		**20** [b]	H_2O	3,010,000	T = 35 °C
	N_2	1,080			n-butanol	16,200	
**9**	H_2	72,100			Biomass	5,460	
	CO_2	144,000			Acetate	5,420	
	N_2	215,200			Organics	3,290	
**10**	H_2	62,000			Na_2SO_4	76,640	
	CO_2	37,000		**21**	Biomass	5,460	
	N_2	214,000		**22**	H_2O	3,010,000	T = 35 °C
**11**	H_2	313			n-butanol	16,200	
	CO_2	187			Acetate	5,420	
	N_2	1,080			Organics	3,290	
**12**	H_2O	3,010,000			Na_2SO_4	76,640	
**13**	NH_3	1,400		**23**	Mesitylene	1,480	
	H_3PO_4	674		**24**	Mesitylene	6,030,000	T = 30 °C
	$MgSO_4$	725		**25**	H_2O	3,010,000	T = 30 °C
**14**	H_2O	3,010,000			n-butanol	162	
	NH_3	1,400			Acetate	5,420	
	H_3PO_4	674			Na_2SO_4	76,600	
	$MgSO_4$	725			Organics	3,290	
**15**	NaOH	42,900		**26**	H_2O	3,010,000	
					Na_2SO_4	76,600	

Stream No.	Components	Flow Rates (kg/h)	Properties	Stream No.	Components	Flow Rates (kg/h)	Properties
27	Mesitylene	6,030,000	T = 30 °C	30	Mesitylene	1,400	T = 83 °C
	n-butanol	16,000			n-butanol	460	
	H_2O	1,410			H_2O	1,330	
28	Mesitylene	6,030,000	T = 148 °C	31	n-butanol	15,500	T = 115 °C
	n-butanol	16,000			Mesitylene	79	
	H_2O	1,410			H_2O	79	
29	Mesitylene	6,030,000	T = 148 °C	32	Mesitylene	6,030,000	T = 164 °C
	n-butanol	15,500		33	Mesitylene	6,030,000	T = 30°C
	H_2O	79					

[a] Pressure (P) and temperature (T) of each stream are assumed to be ambient, unless otherwise stated.

[b] The second bioreactor is also fed oxygen, produced by the electrolyzer; this is omitted from the PFD for ease of visualization.

Table S11: Base-case material flows for Knallgas bacteria-based DAC-EMP process (stream labels shown in Figure S7)

Stream No.	Components	Flow Rates (kg/h)	Properties [a]
**1**	Air	161,000,000	
	CO_2	97,700	
**2**	Air	161,000,000	P = 133 Pa
	CO_2	97,700	(guage)
**3**	Air	161,000,000	
	CO_2	49,100	
**4**	CO_2	48,600	T= 120 ºC
	N_2	492	P = 0.1 atm
**5**	CO_2	48,600	T = 120 ºC
	N_2	492	P = 2 atm
**6**	H_2O	92,400	
**7**	H_2	10,300	
	O_2 [b]	31,700	
**8**	H_2	10,300	
	CO_2	48,600	
	N_2	492	
	O_2	31,700	
**9**	H_2	78,800	
	CO_2	224,000	
	N_2	98,400	
	O_2	244,000	
**10**	H_2	68,500	
	CO_2	175,000	
	N_2	97,900	
	O_2	212,000	
**11**	H_2	344	
	CO_2	879	
	N_2	492	
	O_2	1,070	
**12**	H_2O	1,610,000	
**13**	NH_3	885	
	H_3PO_4	425	
	$MgSO_4$	388	

Stream No.	Components	Flow Rates (kg/h)	Properties
**14**	H_2O	1,610,000	
	NH_3	885	
	H_3PO_4	425	
	$MgSO_4$	388	
**15**	H_2O	1,610,000	T = 30 ºC
	n-butanol	16,100	
	Biomass	5,420	
	Organics	3,270	
**16**	Biomass	5,420	
**17**	H_2O	1,610,000	T = 30 ºC
	n-butanol	16,100	
	Organics	3,270	
**18**	Mesitylene	878	
**19**	Mesitylene	3,230,000	T = 30 ºC
**20**	Mesitylene	3,230,000	T = 30 ºC
	H_2O	758	
	n-butanol	15,900	
**21**	H_2O	1,610,000	
	Organics	3,270	
	n-butanol	161	
**22**	Mesitylene	3,230,000	T = 148 ºC
	H_2O	758	
	n-butanol	15,900	
**23**	Mesitylene	799	T = 83 ºC
	butanol	373	
	H_2O	679	
**24**	Mesitylene	3,230,000	T = 148 ºC
	H_2O	79	
	n-butanol	15,500	
**25**	Mesitylene	3,230,000	T = 164 ºC
**26**	Mesitylene	3,230,000	T = 30 ºC
**27**	n-butanol	15,500	T = 115 ºC
	Mesitylene	79	
	H_2O	79	

[a] Pressure (P) and temperature (T) of each stream are assumed to be ambient, unless otherwise stated.

[b] Only some of the oxygen produced by the electrolyzer is added to the H_2/CO_2 stream, while the rest is vented.

Supporting References

1. Abel, A.J.; Adams, J.D.; Clark, D.S. A comparative life cycle analysis of electromicrobial production systems. *Energy Environ. Sci.* **2022**,*15*, 3062–3085. DOI: 10.1039/D2EE00569G.
2. Fast, A.G.; Papoutsakis, E.T. Stoichiometric and energetic analyses of non-photosynthetic CO_2-fixation pathways to support synthetic biology strategies for production of fuels and chemicals. *Curr. Opin. Chem. Eng.* **2012**, *1*, 380–395. DOI: 10.1016/j.coche.2012.07.005.
3. Tracy, B.P.; Jones, S.W.; Fast, A.G.; Indurthi, D.C.; Papoutsakis, E.T. *Clostridia*: the importance of their exceptional substrate and metabolite diversity for biofuel and biorefinery applications. *Curr. Opin. Biotechnol.* **2012**, *23*, 364–381. DOI: 10.1016/j.copbio.2011.10.008.
4. Vasconcelos, J.M.T.; Orvalho, S.C.P.; Rodrigues, A.M.A.F.; Alves, S.S. Effect of Blade Shape on the Performance of Six-Bladed Disk Turbine Impellers. *Ind. Eng. Chem. Res.* **2000**, *39*, 203–213. DOI: 10.1021/ie9904145.
5. Meraz, J.L.; Dubrawski, K.L.; El Abbadi, S.H.; Choo, K.H.; Criddle, C.S. Membrane and Fluid Contactors for Safe and Efficient Methane Delivery in Methanotrophic Bioreactors. *J. Environ. Eng.* **2020**, *146*. DOI: 10.1061/(ASCE)EE.1943-7870.0001703.
6. McDonald, T.M.; Lee, W.R.; Mason, J.A.; Wiers, B.M.; Hong, C.S.; Long, J.R. Capture of Carbon Dioxide from Air and Flue Gas in the Alkylamine-Appended Metal–Organic Framework mmen-Mg_2(dobpdc). *J. Am. Chem. Soc.* **2012**,*134*, 7056–7065. DOI: 10.1021/ja300034j.
7. Darunte, L.A.; Sen, T.; Bhawanani, C.; Walton, K.S.; Sholl, D.S.; Realff, M.J.; Jones, C.W. Moving Beyond Adsorption Capacity in Design of Adsorbents for CO_2 Capture from Ultradilute Feeds: Kinetics of CO_2 Adsorption in Materials with Stepped Isotherms. *Ind. Eng. Chem. Res.* **2019**, *58*, 366–377. DOI: 10.1021/acs.iecr.8b05042.
8. Casas, N.; Schell, J.; Pini, R.; Mazzotti, M. Fixed bed adsorption of CO_2/H_2 mixtures on activated carbon: experiments and modeling. *Adsorption* **2012**, *18*, 143–161. DOI: 10.1007/s10450-012-9389-z.
9. Sinha, A.; Darunte, L.A.; Jones, C.W.; Realff, M.J.; Kawajiri, Y. Systems Design and Economic Analysis of Direct Air Capture of CO_2 through Temperature Vacuum Swing Adsorption Using MIL-101(Cr)-PEI-800 and mmen-Mg_2(dobpdc) MOF Adsorbents. *Ind. Eng. Chem. Res.* **2017**, *56*, 750–764. DOI: 10.1021/acs.iecr.6b03887.
10. Linstrom, P.J.; Mallard, W.G. *NIST Chemistry WebBook, NIST Standard Reference Database Number 69*; National Institute of Standards and Technology, 2001. DOI: 10.18434/T4D303.
11. Engineering ToolBox. *Heat Exchangers - Heat Transfer Coefficients.* https://www.engineeringtoolbox.com/heat-transfer-coefficients-exchangers-d_450.html (accessed 2023-04-01).
12. Ishizaki, A.; Tanaka, K. Batch culture of *Alcaligenes eutrophus* ATCC 17697T using recycled gas closed circuit culture system. *J. Ferment. Bioeng.* **1990**, *69*, 170–174. DOI: 10.1016/0922-338X(90)90041-T.
13. Takeshita, T.; Ishizaki, A. Influence of Hydrogen Limitation on Gaseous Substrate Utilization in Autotrophic Culture of *Alcaligenes eutrophus* ATCC 17697T. *J. Ferment. Bioeng.* **1996**, *81*, 83–86. DOI: 10.1016/0922-338X(96)83127-8.
14. Stolper, D.A.; Revsbech, N.P.; Canfield, D.E. Aerobic growth at nanomolar oxygen concentrations. *Proc. Natl. Acad. Sci. U.S.A.* **2010**,*107*, 18755–18760. DOI: 10.1073/pnas.1013435107.
15. Groher, A.; Weuster-Botz, D. Comparative reaction engineering analysis of different acetogenic bacteria for gas fermentation. *J. Biotechnol.* **2016**, *228*, 82–94. DOI: 10.1016/j.jbiotec.2016.04.032.
16. Chen, J.; Gomez, J.A.; Höffner, K.; Barton, P.I.; Henson, M.A. Metabolic modeling of synthesis gas fermentation in bubble column reactors. *Biotechnol. Biofuels* **2015**, *8*, 1–12. DOI: 10.1186/s13068-015-0272-5.
17. Wu, X.; Altman, R.; Eiteman, M.A.; Altman, E. Adaptation of *Escherichia coli* to Elevated Sodium Concentrations Increases Cation Tolerance and Enables Greater Lactic Acid Production. *Appl. Environ. Microbiol.* **2014**, *80*, 2880–2888. DOI: 10.1128/AEM.03804-13.
18. Kantzow, C.; Mayer, A.; Weuster-Botz, D. Continuous gas fermentation by *Acetobacterium woodii* in a submerged membrane reactor with full cell retention. *J. Biotechnol.* **2015**, *212*, 11–18. DOI: 10.1016/j.jbiotec.2015.07.020.
19. Andersen, K.B.; von Meyenburg, K. Are Growth Rates of *Escherichia coli* in Batch Cultures

Limited by Respiration? *J. Bacteriol.* **1980**, *144*, 114–123. DOI: 10.1128/jb.144.1.114-123.1980.
20. Noh, M.H.; Lim, H.G.; Woo, S.H.; Song, J.; Jung, G.Y. Production of itaconic acid from acetate by engineering acid-tolerant *Escherichia coli* W. *Biotechnol. Bioeng.* **2018**, *115*, 729–738. DOI: 10.1002/bit.26508.
21. Kotte, O.; Volkmer, B.; Radzikowski, J.L.; Heinemann, M. Phenotypic bistability in *Escherichia coli*'s central carbon metabolism. *Mol. Syst. Biol.* **2014**, *10*, 736. DOI: 10.15252/msb.20135022.
22. Xiao, Y.; Feng, X.; Varman, A.M.; He, L.; Yu, H.; Tang, Y.J. Kinetic modeling and isotopic investigation of isobutanol fermentation by two engineered *Escherichia coli* strains. *Ind. Eng. Chem. Res.* **2012**, *51*, 15855–15863. DOI: 10.1021/ie202936t.
23. Xu, G.; Wu, A.; Xiao, L.; Han, R.; Ni, Y. Enhancing butanol tolerance of *Escherichia coli* reveals hydrophobic interaction of multi-tasking chaperone SecB. *Biotechnol. Biofuels* **2019**, *12*, 164. DOI: 10.1186/s13068-019-1507-7.
24. Perry, R.H.; Green, D.W. *Perry's chemical engineers' handbook*, 8th ed.; McGraw-Hill, 2008.
25. Deutz, S.; Bardow, A. Life-cycle assessment of an industrial direct air capture process based on temperature–vacuum swing adsorption. *Nat. Energy* **2021**, *6*, 203–213. DOI: 10.1038/s41560-020-00771-9.
26. Shiva Kumar, S.; Himabindu, V. Hydrogen production by PEM water electrolysis – A review. *Mater. Sci. Energy Technol.* **2019**, *2*, 442–454. DOI: 10.1016/j.mset.2019.03.002.
27. Zuberi, M.J.S.; Hasanbeigi, A.; Morrow, W. Techno-economic evaluation of industrial heat pump applications in US pulp and paper, textile, and automotive industries. *Energy Effic.* **2023**, *16*, 19. DOI: 10.1007/s12053-023-10089-6.
28. Seider, W.D.; Lewin, D.R.; Seader, J.D.; Widagdo, S.; Gani, R. Ng, K.M. *Product and process design principles : synthesis, analysis, and evaluation*, 4th ed; John Wiley & Sons Inc., 2017.
29. Pivovar, B.S.; Ruth, M.F.; Myers, D.J.; Dinh, H.N. Hydrogen: Targeting $1/kg in 1 Decade. *Electrochem. Soc. Interface* **2021**, *30*, 61–65. DOI: 10.1149/2.F15214IF.
30. van Leeuwen, C.; Mulder, M. Power-to-gas in electricity markets dominated by renewables. *Appl. Energy* **2018**, *232*, 258–272. DOI: 10.1016/j.apenergy.2018.09.217.
31. Humbird, D.; Davis, R.; McMillan, J.D. Aeration costs in stirred-tank and bubble column bioreactors. *Biochem. Eng. J.* **2017**, *127*, 161–166. DOI: 10.1016/j.bej.2017.08.006.
32. Bunch, S.; Cort, K.; Jhonson, E.; Elliott, D.; Stoughton, K.M. *Water and Wastewater Annual Price Escalation Rates for Selected Cities across the United States*; DOE/EE-1670 7781; Pacific Northwest National Lab: Richland, WA, 2017. https://doi.org/10.2172/1413878 (accessed 2023-04-12).
33. Grisales Díaz, V.H.; Olivar Tost, G. Techno-economic analysis of extraction-based separation systems for acetone, butanol, and ethanol recovery and purification. *Bioresour. Bioprocess.* **2017**, *4*, 12. DOI: 10.1186/s40643-017-0142-z.
34. Peccia, J.; Westerhoff, P. We Should Expect More out of Our Sewage Sludge. *Environ. Sci. Technol.* **2015**, *49*, 8271–8276. DOI: 10.1021/acs.est.5b01931.
35. Li, Y.; Kontos, G.A.; Cabrera, D. V, Avila, N.M.; Parkinson, T.W.; Viswanathan, M.B.; Singh, V.; Altpeter, F.; Labatut, R.A.; Guest, J.S. Design of a High-Rate Wastewater Treatment Process for Energy and Water Recovery at Biorefineries. *ACS Sustain. Chem. Eng.* **2023**, *11*, 3861–3872. DOI: 10.1021/acssuschemeng.2c07139.
36. Quon, H.; Sperling, J.; Coughline, K.; Greene, D.; Miara, A.; Akar, S.; Talmadge, M.; Stokes-Draut, J.R.; Macknick, J.; Jiang, S. Pipe Parity Analysis of Seawater Desalination in the United States: Exploring Costs, Energy, and Reliability via Case Studies and Scenarios of Emerging Technology. *ACS ES&T Eng.* **2022**, *2*, 434–445. DOI: 10.1021/acsestengg.1c00270.
37. Davis, R.; Grundl, N.; Tao, L.; Biddy, M.J.; Tan, E.C.D.; Beckham, G.T.; Humbird, D.; Thompson, D.N.; Roni, M.S. *Process Design and Economics for the Conversion of Lignocellulosic Biomass to Hydrocarbon Fuels and Coproducts: 2018 Biochemical Design Case Update*; NREL/TP-5100-71949; National Renewable Energy Laboratory: Golden, CO, 2018. https://doi.org/10.2172/1483234 (accessed 2022-08-06).
38. Humbird, D; Davis, R; Tao, L; Kinchin, C; Hsu, D; Aden, A; Schoen, P; Lukas, J; Olthof, B; Worley, M; Sexton, D; Dudgeon, D. *Process Design and Economics for Biochemical Conversion of Lignocellulosic Biomass to Ethanol: Dilute-Acid Pretreatment and Enzymatic Hydrolysis of Corn Stover*; NREL/TP-5100-47764; National Renewable Energy Laboratory: Golden, CO, 2011. https://doi.org/10.2172/1013269 (accessed 2022-08-06).

39. Short, W.; Packey, D.J.; Holt, T. *A manual for the economic evaluation of energy efficiency and renewable energy technologies*; NREL/TP-462-5173; National Renewable Energy Laboratory: Golden, CO, 1995. https://doi.org/10.2172/35391 (accessed 2023-04-12).
40. Department of the Treasury, Internal Revenue Service. *Publ. 946 Cat. No. 13081F. How To Depreciate Property*, last revised Feb. 2023. https://www.irs.gov/pub/irs-pdf/p946.pdf (accessed 2023-04-12).
41. Jin, H.; Chen, L.; Wang, J.; Zhang, W. Engineering biofuel tolerance in non-native producing microorganisms. *Biotechnol. Adv.* **2014**, *32*, 541–548. DOI: 10.1016/j.biotechadv.2014.02.001.
42. Liu, X.-B.; Gu, Q.-Y.; Yu, X.-B.; Luo, W. Enhancement of butanol tolerance and butanol yield in *Clostridium acetobutylicum* mutant NT642 obtained by nitrogen ion beam implantation. *J. Microbiol.* **2012**, *50*, 1024–1028. DOI: 10.1007/s12275-012-2289-9.
43. Dragosits, M.; Mattanovich, D. Adaptive laboratory evolution – principles and applications for biotechnology. *Microb. Cell Fact.* **2013**, *12*, 64. DOI: 10.1186/1475-2859-12-64.
44. Ahmetović, E.; Martín, M.; Grossmann, I.E. Optimization of Energy and Water Consumption in Corn-Based Ethanol Plants. *Ind. Eng. Chem. Res.* **2010**, *49*, 7972–7982. DOI: 10.1021/ie1000955.
45. Saini, M.; Wang, Z.W.; Chiang, C.-J.; Chao, Y.-P. Metabolic engineering of *Escherichia coli* for production of n-butanol from crude glycerol. *Biotechnol. Biofuels* **2017**, *10*, 173. DOI: 10.1186/s13068-017-0857-2.
